# hUC‐MSCs via β‐NGF Alleviate Cognitive Impairment After Tibial Fracture Surgery by Regulating the STMN2/NMNAT2‐SARM1‐NF‐κB Signaling Pathway

**DOI:** 10.1002/cns.71062

**Published:** 2026-07-29

**Authors:** Kai Wang, Fangjun Wang, Yuhan Zhang, Wenbo Li, Shuai Li, Ying Zhou, Guangkuo Ma, Ziwei Xia, Xueyan Zhou, Liwei Wang

**Affiliations:** ^1^ The Affiliated Xuzhou Clinical College of Xuzhou Medical University Xuzhou Jiangsu Province China; ^2^ Department of Anesthesiology Xuzhou Central Hospital Xuzhou Jiangsu Province China; ^3^ Jiangsu Key Laboratory of New Drug Research and Clinical Pharmacy, College of Pharmacy Xuzhou Medical University Xuzhou Jiangsu Province China

**Keywords:** β‐NGF, dendritic degeneration, mesenchymal stem cells, neuroinflammation, neuronal apoptosis, perioperative neurocognitive disorders

## Abstract

**Background:**

Perioperative neurocognitive disorders (PND) are common postoperative complications, particularly in elderly patients, marked by learning and memory deficits with limited treatment options. Human umbilical cord mesenchymal stem cells (hUC‐MSCs) hold promise due to their neuroprotective and immunomodulatory effects, but their underlying mechanisms remain unclear.

**Methods:**

This study established a PND model in aged mice using tibial fracture intramedullary fixation surgery, followed by intravenous hUC‐MSCs administration. Subsequently, behavioral tests, pathological examination, proteomic analysis, and other experiments were performed to verify the therapeutic effect and underlying mechanism of hUC‐MSCs on PND in mice.

**Results:**

hUC‐MSCs significantly improved cognitive function in PND mice, reduced hippocampal neuronal apoptosis and neuroinflammation, and restored dendritic spine density. Mechanistically, hUC‐MSCs secreted β‐NGF to activate the TrkA signaling pathway, upregulate STMN2 and NMNAT2 expression, and inhibit the SARM1/NF‐κB pathway, thereby alleviating neuroinflammation and dendritic degeneration. Overexpression of SARM1 in the hippocampal CA1 region and β‐NGF knockdown in hUC‐MSCs both reversed the therapeutic effects of hUC‐MSCs, confirming their critical roles.

**Conclusions:**

hUC‐MSCs ameliorate PND pathology through the β‐NGF‐mediated STMN2/NMNAT2‐SARM1‐NF‐κB pathway, offering a novel cell‐based therapeutic strategy for PND. Future research may focus on optimizing the secretory function of hUC‐MSCs or developing small‐molecule drugs targeting β‐NGF to enhance therapeutic efficacy.

## Graphical abstract

This study found that human umbilical cord mesenchymal stem cells secrete β‐NGF, which binds to the TrkA receptor on neurons, upregulates the protein levels of STMN2 and NMNAT2 in mice with perioperative neurocognitive disorders, and thereby downregulates the protein levels of SARM1 and p‐NF‐κB. These changes significantly ameliorate perioperative neurocognitive disorders in mice, reduce neuronal apoptosis, inhibit the progression of neuroinflammation, and alleviate dendritic degeneration.

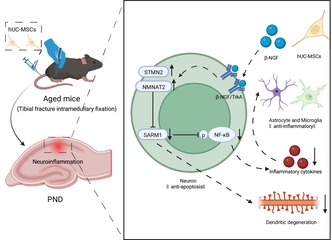

## 1 Introduction

Perioperative neurocognitive disorders (PND) are common anesthesia‐ and surgery‐related complications during the perioperative period, encompassing various forms of cognitive impairment, such as preoperatively diagnosed cognitive decline, postoperative delirium (cognitive dysfunction occurring within 1 week after surgery), delayed neurocognitive recovery (cognitive decline within 30 days postoperatively), and postoperative cognitive dysfunction (cognitive decline diagnosed within 12 months after surgery) [1]. PND is primarily characterized by persistent deficits in learning, memory, and other cognitive domains, leading to prolonged hospitalization and impaired quality of life [2, 3]. Both patients undergoing tibial fracture surgery and elderly patients after surgery have a high incidence of PND. Additionally, the one‐year postoperative mortality rate is significantly increased in patients with PND [4, 5]. The underlying mechanisms may involve acute systemic neuroinflammation triggered by surgical trauma, which subsequently leads to hippocampal neuroinflammation, neuronal apoptosis, and dendritic degeneration. These pathological changes could serve as critical therapeutic targets for PND [6, 7]. However, there is currently a lack of effective drugs in clinical practice for the treatment or prevention of PND. Therefore, exploring PND therapeutic strategies that target mechanisms such as neuroinflammation modulation, neuronal apoptosis inhibition, and dendritic plasticity improvement may represent a promising research direction [8].

Stem cell therapy has stood out as an exceptionally promising intervention for cognitive dysfunction, boasting multi‐target therapeutic capabilities to alleviate cognitive impairment [9, 10]. Mesenchymal stem cells (MSCs), a distinct subtype of adult stem cells, can be isolated from a variety of tissue sources, such as bone marrow, adipose tissue, human umbilical cord (hUC), and placenta [11, 12]. These cells exhibit distinctive therapeutic properties, including anti‐inflammatory, antiapoptotic, and immunomodulatory effects, as well as unique biological properties, particularly their accessibility, robust proliferation ability, and multipotency [13]. Comparative analyses revealed that human umbilical cord‐derived MSCs (hUC‐MSCs) exhibit superior clinical applicability, characterized by enhanced biosafety, preservation of primitive embryonic features, accelerated proliferation kinetics, minimal ethical constraints, and reduced procedural invasiveness. Furthermore, the robust secretory profile of neurotrophic factors positions hUC‐MSCs as optimal candidates for therapeutic applications [14]. Substantial preclinical and clinical evidence has established the efficacy of MSC‐based therapies in ameliorating cognitive deficits associated with neurodegenerative disorders, particularly Alzheimer's and Parkinson's diseases [12, 14, 15, 16, 17]. Given the shared pathological features of cognitive impairment and significant quality‐of‐life deterioration observed in PND, Alzheimer's disease (AD) and Parkinson's disease (PD), investigating the potential therapeutic benefits of hUC‐MSCs for PND represents a compelling and clinically relevant research direction.

Through proteomic analysis, we identified a differentially expressed protein, stathmin‐2 (STMN2), which is a neuron‐specific microtubule‐binding protein involved in neurite and axonal regeneration [18]. STMN2 cooperatively suppresses sterile alpha and Toll/interleukin‐1 receptor motif‐containing 1 (SARM1) expression alongside nicotinamide nucleotide adenylyltransferase 2 (NMNAT2) [19]. SARM1 is a regulator of calpain activity that has been implicated in cognitive impairment. Functioning as a Toll/interleukin‐1 receptor adaptor protein, SARM1 is critically involved in neuronal degeneration, with increased activity correlating with elevated apoptotic signaling [20]. Accumulating evidence demonstrates that SARM1 promotes Wallerian axonal degeneration [21]. Notably, recent studies have revealed its additional role in mediating dendritic degeneration [22], suggesting that SARM1 downregulation may be a promising therapeutic target for PND to alleviate dendritic pathology and restore neuronal function. Furthermore, SARM1 is significantly involved in neuroinflammatory processes. As a member of the MyD88‐like adaptor protein family, SARM1 facilitates protein complex assembly following Toll‐like receptor stimulation, serving as a signaling component that regulates Nuclear Factor kappa‐light‐chain‐enhancer of activated B cells (NF‐κB) activation [23, 24]. The NF‐κB pathway is closely linked to a range of central nervous system (CNS) disorders and serves a pivotal function in neuroinflammation [25, 26]. This pathway can be activated by diverse extracellular signals, including infections, inflammatory cytokines, and cellular stress, ultimately triggering the production of pro‐inflammatory cytokines, chemokines, and inducible enzymes that collectively drive neuroinflammatory responses [27]. Based on these findings, we propose the first hypothesis that hUC‐MSCs may ameliorate PND progression by modulating STMN2 to suppress SARM1 activity, thereby attenuating neuroinflammation, neuronal apoptosis, and dendrite degeneration.

Notably, hUC‐MSCs represent a complex biological entity capable of secreting multiple neurotrophic factors and chemokines, including interleukin 6 (IL‐6), vascular endothelial growth factor A (VEGF‐A), and β‐nerve growth factor (β‐NGF) [28]. A pivotal question remains: which core functional factor mediates the critical role of hUC‐MSCs in regulating cognitive dysfunction? Among these secreted factors, NGF binds to tyrosine receptor kinase A (TrkA) to form the NGF/TrkA signaling complex, which is subsequently transported to neuronal cell bodies. This pathway promotes neuronal survival, differentiation, maintenance, and neurite outgrowth, while demonstrating significant cognitive‐enhancing effects [29, 30]. Importantly, studies have reported markedly reduced NGF levels in the hippocampal tissue of PND mouse models [31]. Furthermore, NGF upregulation has been shown to induce the expression of the neurite‐associated protein STMN2 [32]. Hence, we propose our second hypothesis that hUC‐MSCs exert their therapeutic effects by secreting NGF to activate the NGF/TrkA signaling pathway, thereby stimulating STMN2 production and ultimately mitigating PND pathogenesis and progression.

## 2 Methods

### 2.1 Animals

C57BL/6J mice (male, 16–18 months old) were purchased from GemPharmatech Co. Ltd. (Nanjing, China) and raised at the Animal Center of the Xuzhou Medical University. All animals were housed in climate‐controlled chambers set to 23°C ± 2°C with a relative humidity of 45% ± 5%, under a 12 h light/12 h dark cycle. Prior to the experimental procedures, the mice were given standard rodent diet and water ad libitum and allowed a one‐week acclimation period. The experimental protocol was reviewed and approved by the Xuzhou Animal Care and Use Committee (approval No. 202504T033) and carried out in compliance with the Institutional Animal Care and Use Guidelines of Xuzhou Medical University.

### 2.2 hUC‐MSCs Preparation, Characterization, and Differentiation

hUC‐MSCs were obtained from the Cyagen Biosciences Inc. (Cyagen, China, HUXUC‐01001). The cells were cultured in 15 mL serum‐free medium (Cyagen, China, HUXUC‐90062) in T75 cell culture flasks. Upon reaching approximately 80% confluence, the cells were passaged. Fifth‐passage (P5) hUC‐MSCs were used for subsequent experiments.

P5 hUC‐MSCs were digested with trypsin (Beyotime, China, C0201‐100 mL) and centrifuged. Subsequently, each sample containing 1 × 10^6^ cells was resuspended in 100 μL cold phosphate‐buffered saline (PBS) (Servicebio, China, G4202‐500ML), followed by staining with the following antibodies: FITC‐conjugated antihuman CD29 (Invitrogen, USA, 11–0299‐41), PE‐conjugated antihuman CD44 (Proteintech, China, PE‐65063), APC‐conjugated antihuman CD90 (Invitrogen, USA, 17–0909‐41), FITC‐conjugated antihuman CD34 (Proteintech, China, FITC‐65183), and PE‐conjugated antihuman CD45 (Proteintech, China, PE‐65109). After incubating the cells for 30 min, they were washed and resuspended in 200 μL PBS. A flow cytometer (BD Biosciences, USA, FACSCelesta) was used for cell analysis, and 10,000 events were collected for each sample. Data were analyzed using FlowJo software (BD Biosciences, USA, v.10.8.1).

To verify the adipogenic, osteogenic, and chondrogenic differentiation potential of hUC‐MSCs, the following differentiation assays were performed.

Adipogenic Differentiation: hUC‐MSCs were cultured in 6‐well plates using the aforementioned serum‐free medium. Upon reaching approximately 90% confluence, the cells were induced with hUC‐MSCs Adipogenic Differentiation Induction Complete Medium from the Human Umbilical Cord Marrow Mesenchymal Stem Cells Adipogenic Differentiation Kit (Hysigen, China, UCHX‐D102R) for 3 days, followed by hUC‐MSCs Adipogenic Differentiation Maintenance Complete Medium for 1 day. This induction‐maintenance cycle was repeated for a total of 14 days. Subsequently, the medium was aspirated, and the cells were washed with PBS, fixed in 4% paraformaldehyde for 30 min, and rinsed twice with PBS. A working Oil Red O solution was prepared by mixing Oil Red O stock solution and PBS at a ratio of 3:2. The fixed cells were stained with the working Oil Red O solution for 30 min at room temperature. After removing the staining solution and washing twice with PBS, fat droplets were observed under a light microscope (Olympus, Japan, BX43F).

Osteogenic Differentiation: hUC‐MSCs were cultured in 6‐well plates using the aforementioned serum‐free medium. Upon reaching approximately 90% confluence, the cells were induced with hUC‐MSCs Osteogenic Differentiation Complete Medium from the Human Umbilical Cord Marrow Mesenchymal Stem Cells Osteogenic Differentiation Kit (Hysigen, China, UCHX‐D101R). The medium was replaced every 3 days. After 21 days of induction, the medium was aspirated, and the cells were washed with PBS, fixed in 4% paraformaldehyde for 30 min, and rinsed twice with PBS. The cells were then stained with 2 mL Alizarin Red S staining solution for 5 min. Following removal of the stain and two PBS washes, mineralized matrices were examined under a light microscope (Olympus, Japan, BX43F).

Chondrogenic Differentiation: hUC‐MSCs were adjusted to a density of 1 × 10^7^ cells/mL. A 20‐μL aliquot of the cell suspension was spotted in the center of each well in a 24‐well plate and incubated for 3 h to allow cell attachment. Subsequently, 1 mL of chondrogenic induction medium (Hysigen, China, UCHX‐D203) was added to each well. The induction medium was refreshed every 3 days. After 28 days of culture, the medium was aspirated, and the cells were washed with PBS, fixed in 4% paraformaldehyde for 30 min, and rinsed twice with PBS. The pellets were stained with 1 mL Alcian Blue staining solution for 30 min. After removal of the staining solution and two PBS washes, proteoglycans were visualized under a light microscope (Olympus, Japan, BX43F).

### 2.3 Cell Transfection

β‐NGF‐siRNA was designed and synthesized by Sangon Biotech. The β‐NGF‐siRNA Forward: 5′‐CGACUCACACCUUUGUCAAGG‐3′, Reverse: 5′‐UUGACAAAGGUGUGAGUCGUG‐3′, NC‐siRNA Forward: 5′‐UUCUCCGAACGUGUCACGUTT‐3′ Reverse: 5′‐ACGUGACACGUUCGGAGAATT‐3′. hUC‐MSCs were cultured in T75 cell culture flasks until they reached 70%–80% confluence. Then, siRNA, serum‐free culture medium, and transfection reagent siRNA‐Mate Plus (GenePharma, China, G04026) were diluted in the appropriate proportions. The siRNA mixture was added to the cells in T75 cell culture flasks. The cells were transfected for 2 days, and then β‐NGF protein expression was assessed.

### 2.4 Cell Model and Cell Counting Kit‐8 (CCK‐8) Assay

To create a neuroinflammation model in vivo, we employed a Transwell coculture system (BDBIO, China, H805017) to evaluate hUC‐MSCs‐based therapy. BV2 or HT‐22 cells (4 × 10^5^ cells/well) were seeded in 24‐well plates using DMEM (KeyGEN, China, KGL1206‐500), with or without 1 μg/mL LPS (Sigma, USA, L2630) pretreatment for 24 h. After medium replacement, 3 × 10^4^ hUC‐MSCs were plated in Transwell inserts containing complete hUC‐MSCs growth medium and allowed to adhere. The transwells were then transferred to wells containing LPS‐pre‐treated or untreated BV2/HT‐22 cells for 24 h coculture prior to flow cytometry analysis.

To verify whether β‐NGF can directly promote the expression of STMN2 and NMNAT2 proteins, HT‐22 cells were seeded into 24‐well plates and 96‐well plates. Cells were treated with recombinant human β‐NGF (50 ng/mL) (Abclonal, China, RP01792) alone or in combination with the TrkA inhibitor GW‐441756 (100 nM) (MCE, USA, HY‐18314) for 24 h. Cells in 24‐well plates were harvested for subsequent Western blot analysis. CCK‐8 solution (beyotime, China, C0037) was added to 96‐well plates at 37 ℃ for 1 h, and A450 was measured using a microplate reader to evaluate drug‐induced cytotoxicity.

### 2.5 PND Mouse Model and hUC‐MSCs Transplantation

The tibial fracture intramedullary fixation procedure was performed under isoflurane anesthesia. Anesthesia was induced with 4% isoflurane until the loss of righting reflex, followed by maintenance with 2% isoflurane throughout the surgery. The surgical site was shaved and disinfected with povidone‐iodine. After exposing the tibia, mid‐shaft osteotomy was performed, followed by insertion of a Kirschner wire from the tibial plateau to achieve fracture reduction. The skin was sutured, and 2% lidocaine solution was administered locally for postoperative analgesia while maintaining isoflurane anesthesia. The total surgical duration was approximately 60 min. After surgery, X‐ray was used to observe the placement of the Kirschner wires and determine whether they were positioned intramedullary (Figure S1). A total dose of 1 × 10^6^ hUC‐MSCs in 200 μL PBS per mouse was slowly injected into the mice through tail vein injection after postoperative 30 min.

In tracing studies, DiL‐labeled (APExBIO, USA, B8804) hUC‐MSCs (1 × 10^6^ cells) were delivered via injection. 24 h postinjection, brain sections were prepared and analyzed by confocal microscopy (Olympus, Japan, Olympus FV1000) to determine hUC‐MSCs localization.

### 2.6 Stereotaxic Injection of Adeno‐Associated Virus (AAV)

The AAV 2/9 vector overexpressing SARM1 (HBAAV2/9‐m‐Sarm1‐3xflag‐mcherry) was designed and constructed by Hanbio Biotechnology (Shanghai, China). Wild‐type mice received an intraperitoneal injection of 1% sodium pentobarbital (50 mg/kg) and were then fixed in a stereotaxic instrument (RWD Life Science, China). A total of 2 μL of SARM1‐overexpressing AAV (HBAAV2/9‐m‐Sarm1‐3xflag‐mcherry) or negative control AAV (HBAAV2/9‐CMV‐mcherry) was microinjected into bilateral hippocampal CA1 regions (AP: −2.3 mm; ML: ±1.6 mm; DV: −1.7 mm). The body temperature was maintained throughout the procedure using a thermostatic heating pad. After the injection was administered, the needle was retained in situ for 10 min to ensure the adequate diffusion of the virus. Behavioral tests and model establishment were performed after a 4‐week period of viral expression.

### 2.7 In Vitro and In Vivo Biofluorescent Imaging

After trypsinization of 1 × 10^6^ hUC‐MSCs, the cells were incubated with 5 μg/mL DiR (APExBIO, USA, B8806) in PBS for 30 min. Following centrifugation, the cells were washed twice with 1 mL PBS and centrifuged again. The supernatant was removed, and the cells were resuspended in 200 μL PBS to prepare a cell suspension containing 1 × 10^6^ DiR‐labeled hUC‐MSCs. The suspension was administered to the mice via tail vein injection.

By IVIS Spectrum (Revvity, USA, IVIS Lumina S5), in vivo imaging was performed at seven time points postinjection: 1 h, 12 h, 1 day, 2 days, 3 days, 5 days, and 7 days. For ex vivo imaging at 24 h, mice were sacrificed and the brains were collected. Prior to imaging, the mice were anesthetized with isoflurane and depilated to minimize autofluorescence. The excitation and emission wavelengths were set at 754 and 778 nm, respectively. Longitudinal changes in fluorescence intensity were recorded after injection. The data were analyzed using Living Image software (PerkinElmer, USA, v.4.4) to evaluate the relative fluorescence signal intensity in the regions of interest, with the minimum signal adjusted to the autofluorescence background levels.

### 2.8 Evans Blue Assay

Mice were injected with EB dye (1% EB dye solution in saline, 4 mL/kg) (Beyotime, China, ST3273) via tail vein and sacrificed 2 h postinjection. The animals were transcardially perfused with ice‐cold PBS until the effluent became clear. The hippocampi were dissected and immersed in 500 μL of formamide (Sigma, USA, F9037) for 72 h at room temperature. After centrifugation at 10,000 × g for 10 min at 4°C, the supernatant absorbance was measured at 620 nm using a microplate reader (BioTek, USA, Synergy H1) with formamide as the blank. EB concentration was calculated based on a standard curve.

### 2.9 Open Field Test (OFT)

The open field test was conducted to evaluate postoperative locomotor activity in mice. The experimental setup consisted of a large open arena (40 cm × 40 cm × 40 cm), and each mouse was placed in the lower right corner for 5 min. Movement distance was recorded using the Any‐maze tracking system (Stoelting, USA, v.6.1). To eliminate residual olfactory cues that might influence subsequent mice, the arena was thoroughly cleaned with 75% ethanol after each test session.

### 2.10 Y‐Maze Test

A Y‐maze apparatus was used to assess the spatial learning and memory abilities of the mice. This maze comprised three white opaque acrylic arms, with respective length, width, and height of 35 cm, 7 cm, and 15 cm. During the test, each mouse was placed at the maze center and permitted to explore all three arms freely for 5 min. Mouse behaviors were recorded and analyzed using an Any‐maze tracking system (Stoelting, USA, v6.1). The alternative correction rate was calculated via the formula: Alternative correction rate (%) = [number of spontaneous alternations]/[total number of arm entries−2] × 100. Here, a spontaneous alternation was defined as the mouse entering a distinct arm in each of three consecutive arm entries.

### 2.11 Novel Object Recognition Test (NOR)

The cognitive memory capacity of the mice was evaluated using the novel object recognition test. The apparatus consisted of a white opaque cubic chamber (40 cm × 40 cm × 40 cm). The experiment comprised two distinct phases: The initial 2 days served as the habituation phase, during which each mouse was placed in the apparatus facing a wall and allowed to freely explore for 10 min daily to acclimatize to the environment. On the third day, during the observation phase, two identical objects matching in shape, size, and color were positioned symmetrically within the chamber. Each mouse was introduced into the apparatus with its back to the objects and allowed 10 min of free exploration. Following the training session, the mice were placed back in their home cages for a 1 h resting period. Subsequently, one object was then replaced with a novel one that varied in both shape and color. The mice were then reintroduced into the test apparatus for a 10 min exploration session. After completion, all the mice were returned to their home cages. Between trials, the apparatus was thoroughly sanitized with 75% ethanol to remove olfactory cues that might have influenced the test outcomes. The exploration time of each object was recorded only when the mice touched the object with their nose and/or directed their nose to the object within 2 cm. The novelty preference index was calculated as follows: Novel Object Preference (%) = Time spent exploring the novel object/Total time spent exploring both objects.

### 2.12 Flow Cytometry

To investigate the effect of hUC‐MSCs on the polarization of BV2 cells. BV2 cells subjected to different treatments were co‐incubated with APC‐conjugated anti‐mouse CD206 (Biolegend, USA, 141707) and PE‐conjugated anti‐mouse CD86 (Biolegend, USA, 159203) antibodies under dark conditions at 4°C for 25 min. After incubation, the cells were washed and resuspended in 100 μL PBS, followed by analysis using a flow cytometer (BD Biosciences, USA, FACSCelesta). A total of 10,000 events were collected per sample.

To investigate the effect of hUC‐MSCs on apoptosis in HT‐22 cells. HT‐22 cells receiving various treatments were processed according to the manufacturer's protocol of the apoptosis detection kit (APExBIO, USA, K2003). Flow cytometry was performed for 10,000 events per sample. All data were analyzed using FlowJo software (BD Biosciences, USA, v.10.8.1).

### 2.13 Immunofluorescence Staining

The intact brains of mice were removed through anesthesia and perfusion. After embedding in OCT compound (SAKURA, USA, 4583) and freezing, 8 μm coronal sections were prepared using a cryostat. Brain slices were subsequently fixed in 4% paraformaldehyde for 10 min and incubated with 0.25% Triton X‐100 (Biosharp, China, BS084) for 10 min, followed by blocking with 10% normal goat serum containing 0.1% Triton X‐100 for 60 min at room temperature. Afterwards, the brain sections were incubated overnight at 4°C with the following primary antibodies: rabbit anti‐NeuN recombinant antibody (1:200, CST, USA, 24307), rabbit anti‐Iba1 monoclonal antibody (1:500, Proteintech, China, 66,827–1‐Ig), rabbit anti‐GFAP polyclonal antibody (1:200, Proteintech, China, 16,825–1‐AP), rat anti‐CD86 monoclonal antibody (1:100, Abcam, UK, ab119857), rat anti‐CD206 monoclonal antibody (1:100, GeneTex, USA, GTX42263), rabbit anti‐SARM1 polyclonal antibody (1:200, Proteintech, China, 28,625–1‐AP), chicken anti‐map2 recombinant antibody (1:100, Abcam, UK, ab318993). After washing three times in PBS, the brain sections were incubated with secondary antibodies conjugated with Alexa 488 (1:500, Vicmed, China, VA1022), 594 (1:500, Abbkine, China, A23440), or 637 (1:200, Yeasen, China, 34713ES60) for 60 min at room temperature. Slices were mounted using Antifade Mounting Medium containing DAPI (Abcam, UK, ab285390) and visualized with a laser scanning confocal microscope (Olympus, Japan, Olympus FV1000).

### 2.14 Golgi‐Cox Staining

Golgi‐Cox staining was performed using a Golgi‐Cox staining kit (FD NeuroTechnologies, USA, PK401) to examine dendritic spine density. Seven days post‐surgery, the mice were euthanized, and their brains were immediately extracted without perfusion. The brain surfaces were rinsed with double‐distilled water to remove blood contamination. The brains were then immersed in Golgi‐Cox impregnation solution (a 1:1 mixture of solutions A and B) at room temperature under light‐protected conditions for 2–3 weeks, followed by transfer to solution C for an additional week. Coronal sections (100 μm thick) were prepared using a vibratome and mounted onto gelatin‐coated slides. After staining, the sections were cleared in xylene and coverslipped following alcohol dehydration. Neuronal images of the hippocampal CA1 region were captured using a microscope (Olympus, Japan, Olympus BX43F). Quantitative analysis was performed using ImageJ software (NIH, USA, v.1.4.3.67). For each experimental group, neurons from the CA1 region were selected from six mice, and dendritic spine density was calculated in a blinded manner.

### 2.15 TUNEL Staining

Terminal deoxynucleotidyl transferase dUTP nick end labeling (TUNEL) assay (meilunbio, China, MA0224‐2) was performed to investigate apoptosis in the hippocampus after surgery using an in situ cell death detection kit. On postoperative day 3, the mice were subjected to cardiac perfusion with physiological saline followed by 4% paraformaldehyde, after which the brains were collected. The harvested brain tissue was fixed overnight and subsequently dehydrated in 30% sucrose solution for 48 h. Following dehydration, the tissue was sectioned into 8 μm‐thick slices and stained using the TUNEL staining kit. Cell nuclei were counterstained with DAPI before mounting. Apoptotic cells were visualized by confocal microscopy (Olympus, Japan, Olympus FV1000) at excitation wavelengths of 405 and 594 nm.

### 2.16 Human Cytokine Antibody Array Analysis

hUC‐MSCs were expanded to 4 × 10^6^ cells. After 3 days, the supernatant was collected and centrifuged at 10,000 rpm for 10 min at 4°C to remove cellular debris. The supernatant was then concentrated 10‐fold using an ultrafiltration tube (Millipore, Germany, UFC9003). Neurotrophic factor screening was performed using the Human Neuro Discovery Array C1 (RayBiotech, USA, AAH‐NEU‐1‐2) according to the manufacturer's instructions. Membranes were imaged using a ChemiDoc imaging system (Bio‐Rad, USA).

### 2.17 Enzyme‐Linked Immunosorbent Assay (ELISA)

Three days post‐surgery, hippocampal tissues were collected immediately after euthanasia and homogenized on ice in cell lysis buffer supplemented with protease and phosphatase inhibitors. The supernatant was harvested following the manufacturer's instructions, and the concentrations of IL‐1β (HUABIO, China, EM0001), IL‐6 (LIANKE, China, EK206), TNF‐α (HUABIO, China, EM0010), mouse (HUABIO, China, RK00154), and human β‐NGF (Abclonal, China, RK03064) were quantified using ELISA kits. Cytokine levels were normalized to the total protein content and expressed as picograms per milligram of protein (pg/mg).

The hUC‐MSCs‐conditioned supernatant was collected according to the manufacturer's instructions, and the concentrations of β‐NGF (HUABIO, China, RK00154) were quantified using ELISA kits.

### 2.18 Western Blot

Hippocampal tissue was added to lysis buffer (Beyotime, China, P0013B), cut into pieces with scissors, and processed with a grinder. Total protein was separated by SDS‐PAGE and transferred to an NC membrane (Millipore, Germany, HATF00010). The membranes were blocked with protein‐free rapid blocking solution (Servicebio, China, G2052‐500ML) for 15 min and rapidly washed with 1× PBS for 2 min. The NC membranes were incubated overnight at 4°C with the following antibodies: anti‐STMN2 (1:1000, Proteintech, China, 10,586–1‐AP), anti‐NMNAT2 (1:1000, Proteintech, China, 27,698–1‐AP), anti‐SARM1 (1:1000, Proteintech, China, 28,625–1‐AP), anti‐NF‐κB (1:1000, Affinity, China, AF5006), anti‐p‐NF‐κB (1:1000, Affinity, China, AF2006), anti‐β‐NGF (1:1000, HUABIO, China, ET1606‐29), anti‐TrkA (1:1000, abclonal, China, A4147), anti‐p‐TrkA (1:1000, abclonal, China, AP0492), anti‐α‐Tubulin (1:10000, abclonal, China, A6830), anti‐β‐actin (1:10000, Bioworld, USA, AP0060). The membranes were then incubated with secondary antibodies at room temperature. After 1 h, the protein bands were examined using near‐infrared fluorescence imaging technology (LI‐COR, USA, Odyssey CLX). The band intensity was analyzed using ImageJ software (NIH, USA, v.1.4.3.67).

### 2.19 Quantitative Real‐Time PCR

Total RNA was extracted from approximately 30 mg of hippocampal tissue using RNA‐easy Isolation Reagent (Vazyme, China, R701‐01). The RNA concentration was measured by a super‐trace ultraviolet spectrophotometer (Thermo, USA). Total RNA (1 μg) was reverse transcribed. The following primer pairs were used: GAPDH forward 5′‐CTGGAGAAACCTGCCAAGTATG‐3′, reverse 5′‐GGTGGAAGAATGGGAGTTGCT‐3′; STMN2 forward 5′‐GGACTCGGCAGAAGACCTTC‐3′, reverse 5′‐GCAGGCTGTCTGTCTCTCTC‐3′; NMNAT2 forward 5′‐CCGCAATTGAAGGATGTTG‐3′, reverse 5′‐CTCTGGCTCTTGGGATTCTG‐3′; SARM1 forward 5′‐CTATACGTCTGGACGGTGGC‐3′, reverse 5′‐CCAGGTTCAAGATCACGCCT‐3′; NF‐κB forward 5′‐GCAGAAAGAAGACATTGAGGTGTAT‐3′, reverse 5′‐GCGATCATCTGTGTCTGGCA‐3′; MAP4 forward 5′‐TCAGGAGTATCCCGGCAAGA‐3′, reverse 5′‐TTGGTGGTGGCTCCTTGTTT‐3′; Arfgef1 forward 5′‐CAATTCCTCACGCGCTTCTG‐3′, reverse 5′‐TGCATCCAACGGCTTTTCAC‐3′; Rasgrf1 forward 5′‐CGGACAACCCAAAATGGCAA‐3′, reverse 5′‐GACTCCTGCTATGACGCTGT‐3′; β‐NGF forward 5′‐GAGACTCTGTCCCTGAAGCCCA‐3′, reverse 5′‐CCACAGTGATGTTGCGGGTCT‐3′; Relative mRNA expression was normalized to the GAPDH level and calculated by the 2^−ΔΔCT^ method.

### 2.20 Sample Preparation for Proteomic Analysis

About 10 mg of fresh tissue samples (wet weight) were homogenized in 200 μL 8 M urea (with protease inhibitor cocktail). After centrifugation, the soluble proteins were collected and protein concentration was measured by BCA assay. Proteins were reduced by 10 mM TCEP at 56°C for 60 min, and alkylated by 55 mM chloroacetamide (CAA) at room temperature for 30 min in the dark. After dilution to 10 volumes with 50 mM NH_4_HCO_3_ (pH 7.8), proteins were digested overnight at 37°C with trypsin at a 1:50 (enzyme/protein, m/m) ratio. All the digests were desalted by C18‐trap cartridges and lyophilized in SpeedVac. Samples were stored at −80°C until further use.

### 2.21 LC–MS Analysis

Dried peptides from each method were resuspended in 0.1% formic acid at 1 μg/μL. Peptides were separated using an EasyLC system (Thermo Scientific). For separation, a 150 μm inner diameter capillary column was packed to 15 cm with 3 μm, 120 Å pore size C18 particles (Welch Inc., Shanghai, China) in‐house.

Mobile phase A (98% H_2_O + 2% ACN + 0.1% FA) and Mobile phase B (2% H_2_O + 98% ACN + 0.1% FA) were used for gradient separation. A 75 min separation gradient was performed using 5%–10% B (0 − 5 min), 10%–28% B (10–55 min), 28%–40% B (55–58 min), 40%–95% B (58–63 min), and 95%–95% (63–75 min) at a flow rate of 600 nL/min.

Mass Spectrometry analysis was performed using positive mode data‐independent acquisition (DIA) on a Q Exavtive HF MS. MS1 was acquired in Orbitrap mode with 30 k resolution over 350–1500 m/z. The AGC target was set to 1e6 with a maximum injection time of 54 ms. DIA MS2 was acquired with a precursor isolation range of 400–1022 m/z divided into 8 m/z DIA windows resulting in 75 MS2 scans per cycle. HCD collision energy was set to 30% with a default charge state of +2.

Raw files were searched using DIANN 1.8.1 in library‐free mode. The searches used the mouse reference proteome downloaded from UniProt on November 04, 2023.

### 2.22 Statistical Analysis

Statistical analyses were conducted using GraphPad Prism 9.5 software (GraphPad Software Inc., USA, v.9.5.0.). All data were expressed as mean ± standard error of the mean (SEM). Two‐way analysis of variance (two‐way ANOVA) was used for two‐factor comparisons, One‐way analysis of variance (one‐way ANOVA) was used to compare data across multiple groups, and Student's *t*‐test was employed to assess differences between two groups. A *p*‐value < 0.05 was considered statistically significant.

## 3 Results

### 3.1 Morphological and Immunophenotypic Characteristics of hUC‐MSCs and Their Differentiation Potential

P5 hUC‐MSCs exhibited typical fibroblast‐like morphology (Figure S2A). We evaluated the differentiation potential of the hUC‐MSCs. Upon induction with osteogenic, adipogenic, or chondrogenic differentiation media, Alizarin Red staining confirmed the presence of calcium phosphate deposits with extensive extracellular matrix mineralization (Figure S2B). Oil Red O staining demonstrated the formation of lipid droplets, indicating adipogenic differentiation (Figure S2C). Additionally, Alcian Blue staining verified the presence of acidic mucopolysaccharides in chondrogenic tissue (Figure S2D). Furthermore, flow cytometry analysis confirmed that the P5 hUC‐MSCs used in this study were positive for the surface markers CD29, CD44, and CD90, but negative for the hematopoietic stem cell marker CD34 and leukocyte marker CD45 (Figure S2E–I).

These results demonstrated that the hUC‐MSCs used in this study exhibited characteristics consistent with the international definition of MSCs.

### 3.2 hUC‐MSCs Alleviated Surgery‐Induced Learning and Memory Impairments

**FIGURE 1:**
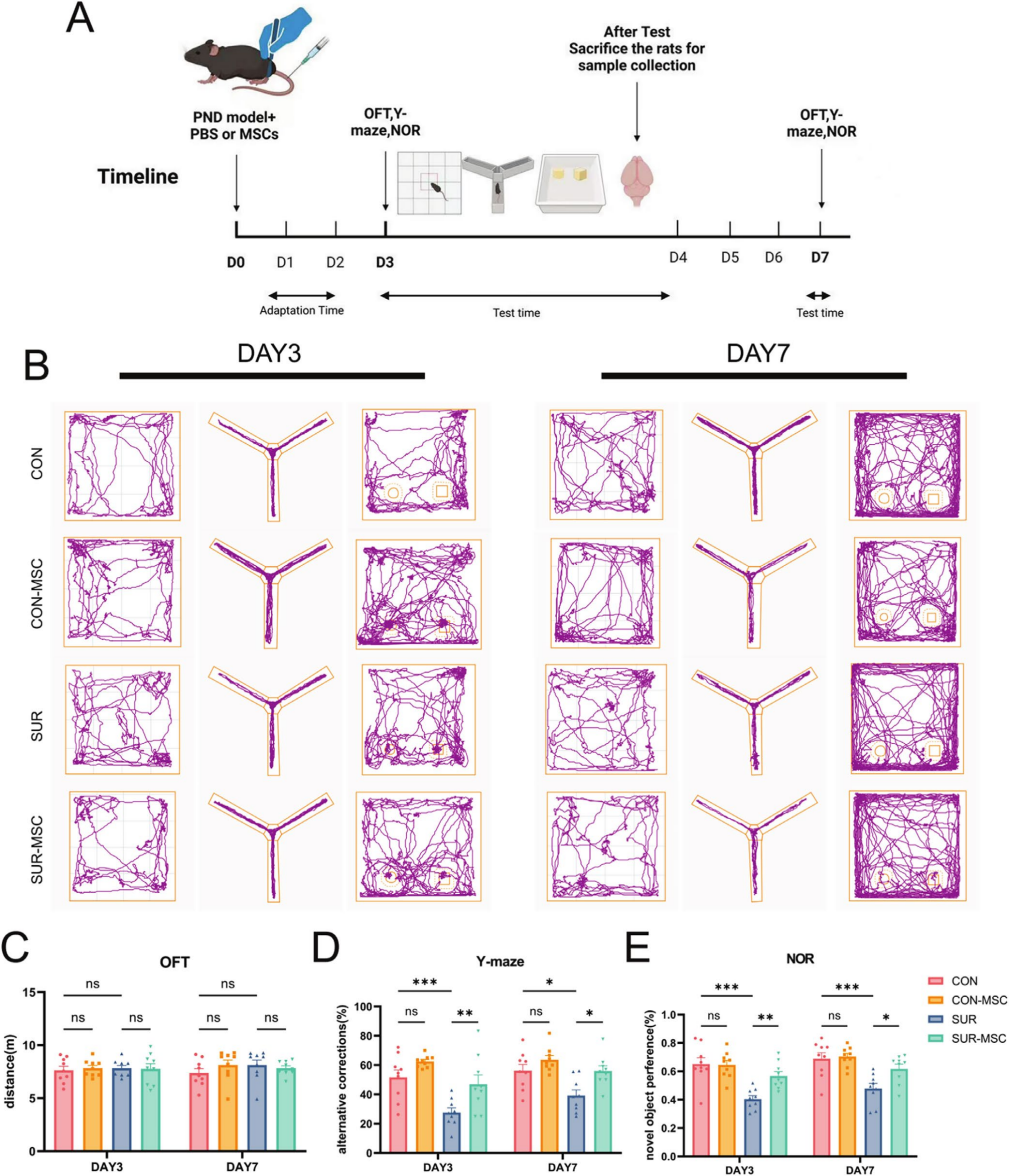
hUC‐MSCs alleviated surgery‐induced learning and memory impairments. (A) Schematic timeline illustrating PND model establishment, hUC‐MSCs treatment, and behavioral assessments including open field test (OFT), Y‐maze test, and novel object recognition test (NOR). (B) Representative movement trajectories of mice in OFT, Y‐maze, and NOR on postoperative days 3 and 7. (C) Total distance traveled by mice in OFT (*n* = 9). (D) Alternative corrections (%) in Y‐maze test (*n* = 9). (E) Novel object preference (%) in NOR (*n* = 9). Data were shown as mean ± SEM. *p < 0.05, **p < 0.01, ***p < 0.001.

To investigate the effects of hUC‐MSCs on tibial fracture intramedullary fixation surgery‐induced cognitive deficits, we conducted a series of behavioral tests on Days 3 and 7 after surgery, including open field, Y‐maze, and novel object recognition tests, to assess motor function and learning/memory capacity in the mice (Figure 1A).

In the open field test, no significant differences in the total travel distance were observed among the CON, CON‐MSC, SUR, and SUR‐MSC groups on days 3 and 7, indicating preserved locomotor activity across all groups 3 days after surgery. This confirmed that motor function did not confound subsequent cognitive assessments (Figure 1B,C). Y‐maze and novel object recognition tests were performed to evaluate cognitive function. On postoperative day 3, the SUR group exhibited significantly reduced spontaneous alternation and novel object preference compared to the CON group, confirming successful establishment of PND following tibial intramedullary fixation (Figure 1B,D,E). Notably, the SUR‐MSC group showed significant improvement in both parameters compared to the SUR group, demonstrating hUC‐MSCs‐mediated mitigation of surgery‐induced cognitive decline. While the same trends persisted on day 7, the intergroup differences were attenuated compared to those on day 3.

These results demonstrated that hUC‐MSCs ameliorated cognitive dysfunction in PND mice induced by fracture fixation surgery.

### 3.3 hUC‐MSCs Demonstrated the Capacity to Transmigrate Across the Compromised Blood–Brain Barrier and Localize to Cerebral Tissues

**FIGURE 2:**
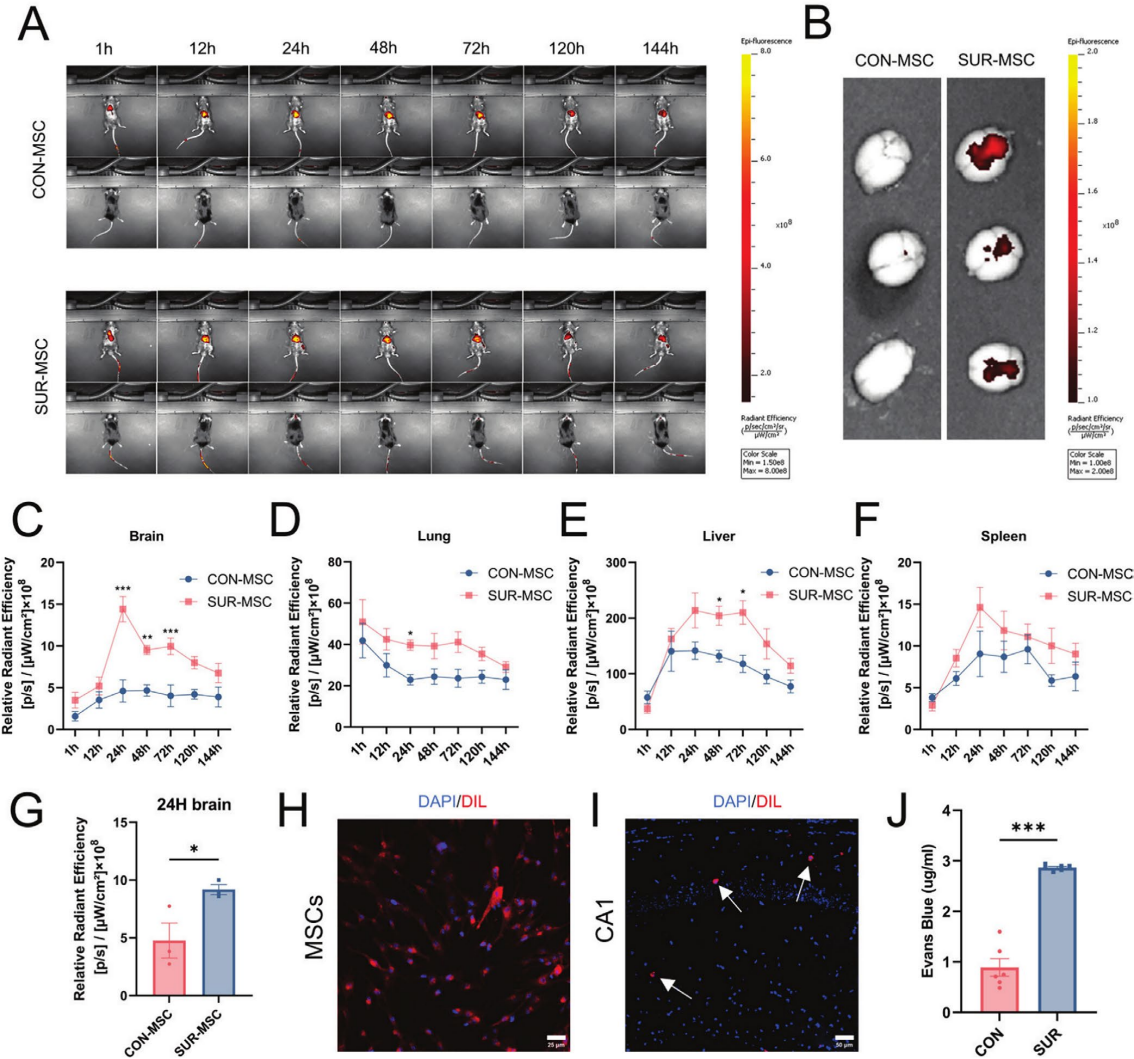
hUC‐MSCs demonstrated the capacity to transmigrate across the compromised blood–brain barrier and localize to cerebral tissues. (A) In vivo fluorescence imaging of representative mice in the CON‐MSC and SUR‐MSC groups over time. (B) Ex vivo fluorescence imaging of brain tissues at 24 h postinjection. Quantitative analysis of relative radiant efficiency in the (C) brain, (D) lung, (E) liver, and (F) spleen in vivo (*n* = 6). (G) Quantitative analysis of relative radiant efficiency in brain tissues at 24 h ex vivo (*n* = 3). (H) Representative fluorescence images showing the DiL labeling of MSCs. (I) Representative fluorescence images showing MSCs localization in the CA1 regions of the hippocampus. (J) Quantitative data of Evans Blue dye extravasation (*n* = 6). Data were shown as mean ± SEM. *p < 0.05, **p < 0.01, ***p < 0.001.

To investigate the mechanism by which hUC‐MSCs improve cognitive function, we tracked the in vivo distribution of hUC‐MSCs using DIR fluorescent labeling. After intravenous injection of DIR‐labeled hUC‐MSCs into CON and SUR group mice, in vivo imaging revealed that within 1 h postinjection, the cells primarily accumulated in the lungs in both groups. By 12 h, the cells were mainly distributed in the liver. The liver maintained high fluorescence signals from 12 to 72 h, while the spleen showed sustained strong signals between 24 and 72 h. Notably, no fluorescence was detected in brain tissue before 24 h. After 24 h, weak signals appeared in the SUR group brains but not in the CON group, indicating limited hUC‐MSC migration to the brain (Figure 2A,C–F). Ex vivo imaging yielded consistent results (Figure 2B,G). Fluorescence imaging confirmed the presence of DiL‐labeled hUC‐MSCs in the damaged hippocampus (Figure 2H,I). These findings suggest that hUC‐MSCs preferentially target injured brain tissue, likely due to blood–brain barrier (BBB) disruption.

Therefore, we subsequently performed Evans Blue extravasation assays to evaluate BBB integrity. Consistent with our hypothesis, Evans blue (EB) leakage and accumulation were observed in the SUR group mice but not in the CON group mice, confirming surgery‐induced BBB disruption (Figure 2J).

These results demonstrated that surgery‐induced cognitive deficits disrupted the BBB, enabling hUC‐MSCs to migrate to the brain, albeit with low engraftment efficiency. This suggested that the therapeutic effects of hUC‐MSCs were likely mediated primarily through paracrine signaling and direct cell–cell interactions.

### 3.4 hUC‐MSCs Alleviate Surgery‐Induced Neuronal Apoptosis and Abnormal Dendritic Morphology

**FIGURE 3:**
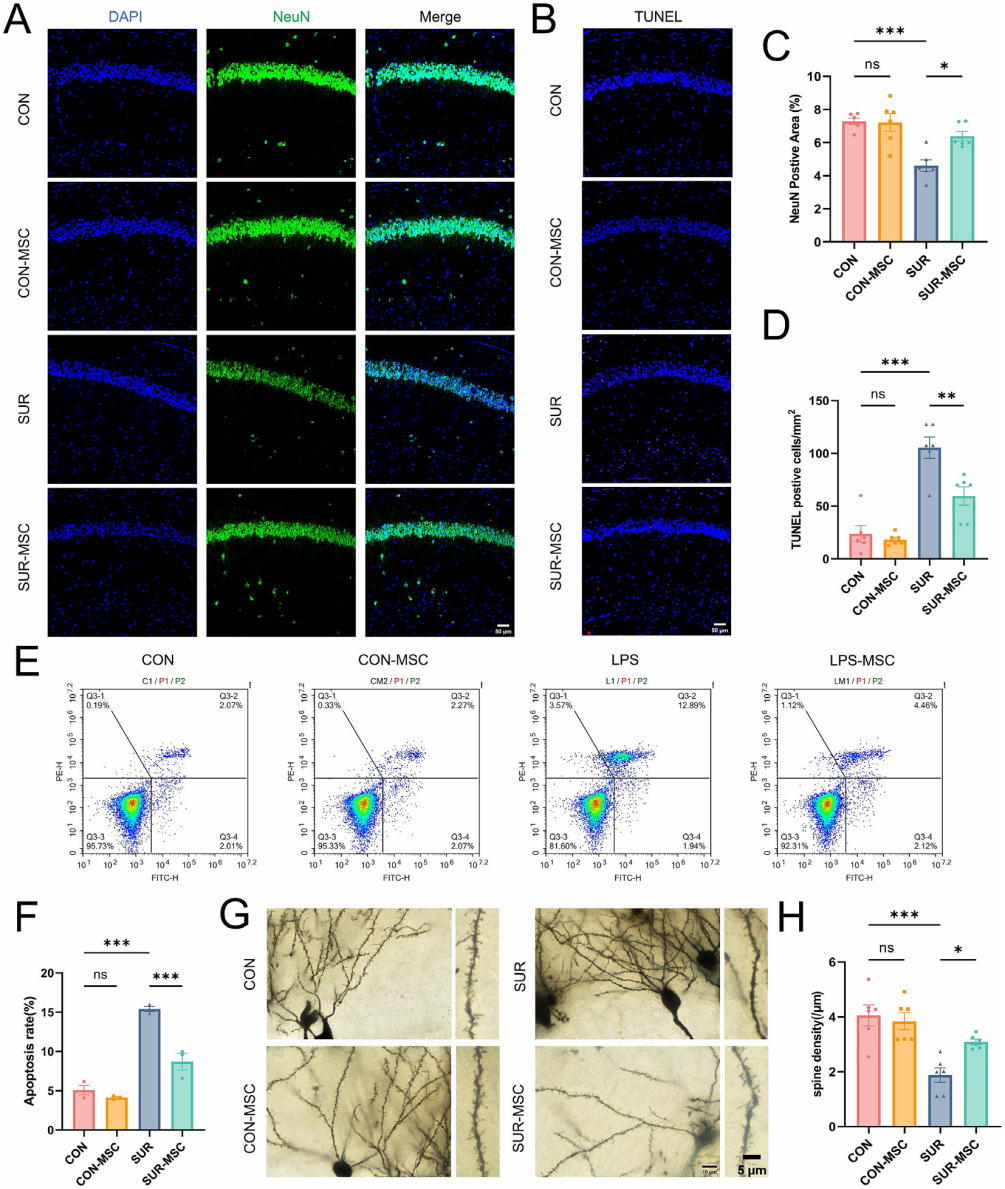
hUC‐MSCs alleviate surgery‐induced neuronal apoptosis and abnormal dendritic morphology. (A) Representative NeuN immunostaining images of the hippocampal CA1 region. (B) Representative TUNEL immunostaining images of the hippocampal CA1 region. (C) Quantitative analysis of the percentage of NeuN positive area in the hippocampal CA1 region (*n* = 6). (D) Quantitative analysis of TUNEL positive cell density in the hippocampal CA1 region (*n* = 6). (E) Flow cytometry analysis of apoptosis in HT22 cells under different treatment conditions. (F) Quantitative analysis of HT22 apoptosis rate under different treatment conditions (*n* = 3). (G) Representative Golgi staining images of the hippocampal CA1 region. (H) Quantitative analysis of dendritic spine density in the hippocampal CA1 region (*n* = 6). Data are presented as mean ± SEM. *p < 0.05, **p < 0.01, ***p < 0.001.

To investigate how hUC‐MSCs improve cognitive function, we examined their effects on neuronal apoptosis, given the well‐established association between neurons and cognitive performance [33, 34]. Immunofluorescence showed that compared to the CON group, the SUR group exhibited increased neuronal apoptosis in the CA1 region, while the SUR‐MSC group showed reduced neuronal apoptosis (Figure 3A,C). TUNEL staining showed that the hippocampal CA1 region exhibited significantly enhanced apoptosis signals in the SUR group, whereas the CON, CON‐MSC, and SUR‐MSC groups displayed markedly fewer apoptotic cells (Figure 3B,D).

To further validate our findings in vitro, we established a neuroinflammatory model using LPS‐stimulated HT‐22 cells to mimic postoperative conditions. Through Transwell coculture with hUC‐MSCs, we observed that compared to the CON group, LPS treatment significantly increased the apoptosis rate of HT‐22 cells, while hUC‐MSCs coculture effectively attenuated LPS‐induced HT‐22 cells apoptosis (Figure 3E,F). Furthermore, as dendritic spine density serves as a sensitive indicator of impaired synaptic plasticity, Golgi‐Cox staining confirmed that surgical intervention induced significant spine loss in the CA1 region, while hUC‐MSCs administration effectively restored normal dendritic morphology (Figure 3G,H).

These results demonstrated that surgical intervention induced both neuronal apoptosis and dendritic spine loss in the hippocampal CA1 region, while hUC‐MSCs treatment effectively attenuated surgery‐induced neuronal death and restored spine density.

### 3.5 hUC‐MSCs Alleviated Surgery‐Induced Neuroinflammation

In addition to neuronal apoptosis and abnormal dendritic morphology, neuroinflammation serves as a key contributor to the pathogenesis of PND [7, 35]. Both surgery and anesthesia can trigger neuroinflammatory responses, primarily mediated by microglia, astrocytes, and pro‐inflammatory cytokines, which serve as key markers of neuroinflammation [36, 37]. To investigate the effect of hUC‐MSCs on neuroinflammation in the mouse brain, inflammatory cytokines in hippocampal tissues were measured. ELISA assays on hippocampal tissue revealed elevated levels of pro‐inflammatory cytokines (IL‐1β, IL‐6, and TNF‐α) in the SUR group compared to the CON group, while hUC‐MSCs administration attenuated this upregulation (Figure S3A). Furthermore, the expression of IBA‐1 and GFAP in the hippocampus was examined. It was found that compared with the CON group, the density of IBA‐1^+^ and GFAP^+^ cells in the CA1 region of the hippocampus significantly increased in the SUR group, and hUC‐MSCs administration reversed this increase (Figure S3B,C). No statistically significant differences were observed in the CA3 or DG regions (Figure S3D–G). Thus, hUC‐MSCs ameliorated surgery‐induced activation of microglia and astrocytes in mice.

**FIGURE 4:**
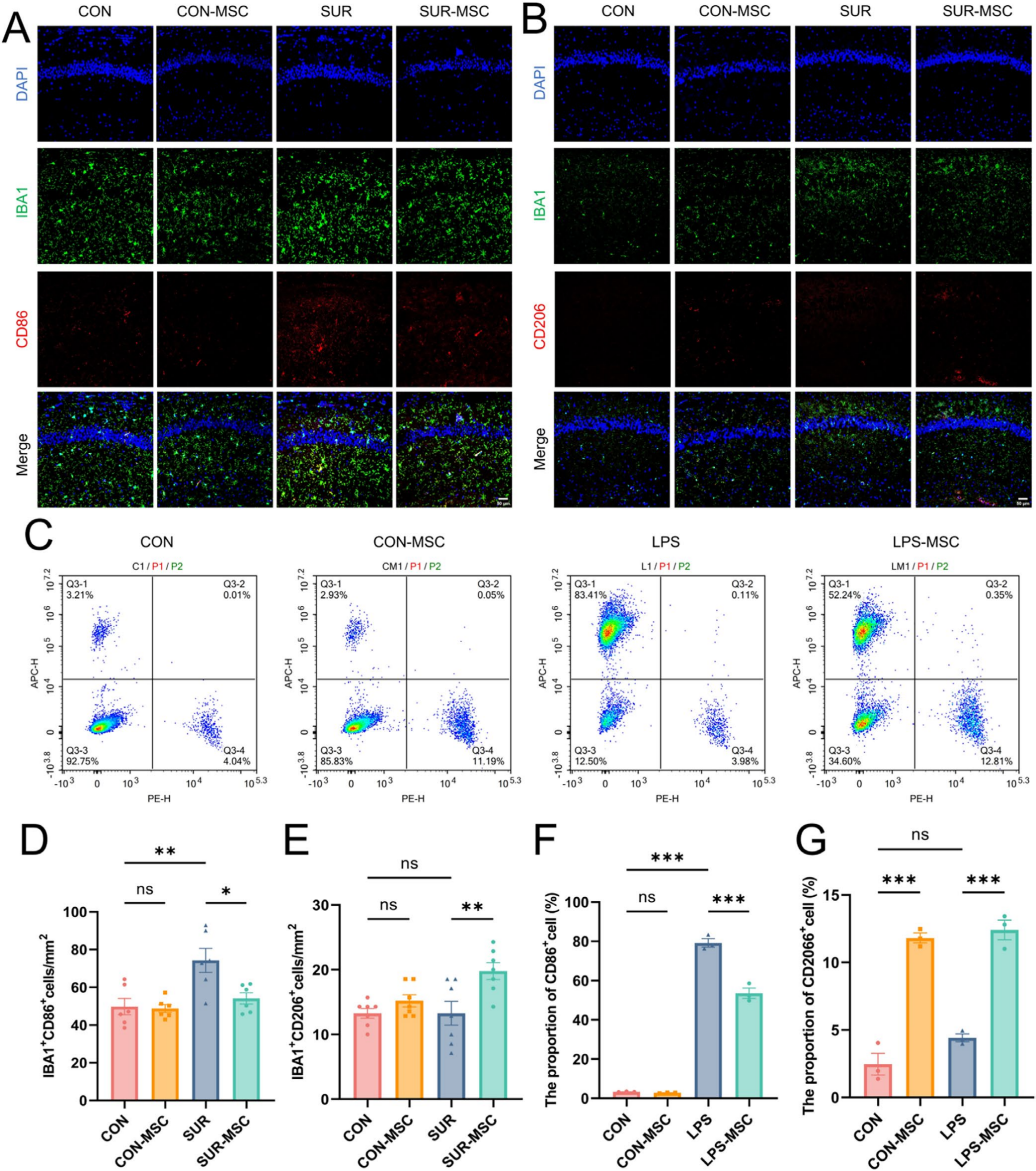
hUC‐MSCs inhibit M1 microglial polarization and promote M2 phenotype switching post‐surgery. (A) Representative IBA1 and CD86 immunostaining images of the hippocampal CA1 region. (B) Representative IBA1 and CD206 immunostaining images of the hippocampal CA1 region. (C) Flow cytometry analysis of BV2 cell polarization status under different treatment conditions. (D) Quantitative analysis of IBA1 + CD86+ cell density in the hippocampal CA1 region (*n* = 6). (E) Quantitative analysis of IBA1 + CD206+ cell density in the hippocampal CA1 region (*n* = 6). (F, G) Quantitative analysis of M1 and M2 cell proportions in BV2 cells under different treatment conditions (*n* = 3). Data are presented as mean ± SEM. *p < 0.05, **p < 0.01, ***p < 0.001.

To explore neuroinflammation in greater depth, research has demonstrated that microglia can become activated and polarized into two distinct phenotypes: the M1 phenotype and the M2 phenotype [38]. M1 microglia are known to exhibit pro‐inflammatory effects, releasing inflammatory cytokines that contribute to brain injury. In contrast, M2 microglia promote CNS repair by secreting anti‐inflammatory cytokines to resolve inflammation [39, 40]. M1 microglia were quantified by counting IBA‐1^+^CD86^+^cells, while M2 microglia were identified as IBA‐1^+^CD206^+^cells. Immunofluorescence results demonstrated that compared to the CON group, the SUR group exhibited a significant increase in IBA‐1^+^CD86^+^cells in the hippocampal CA1 region. However, hUC‐MSCs treatment reduced the number of IBA‐1^+^CD86^+^cells (Figure 4A,D). In contrast, no significant difference was observed in IBA‐1^+^CD206^+^cells between the CON and SUR groups, whereas the SUR‐MSC group showed a marked increase in IBA‐1^+^CD206^+^cells (Figure 4B,E). In vitro, we used BV2 microglial cells treated with LPS to mimic postoperative neuroinflammation. Flow cytometry analysis revealed that compared to the CON group, the LPS group had a significant increase in M1 microglia, while M2 microglia remained unchanged. However, the LPS‐MSC group showed a decrease in M1 microglia and a significant increase in M2 microglia compared to the LPS group (Figure 4C,F,G). Notably, compared with the CON group, the CON‐MSC group significantly increased the number of M2 microglia at the cellular level, whereas no obvious increase was observed in vivo. This discrepancy may be attributed to the fact that hUC‐MSCs are still hindered by the blood–brain barrier, even though the barrier is disrupted to a certain extent after surgery.

These results demonstrate that surgical intervention induces neuroinflammation in the hippocampal CA1 region, and hUC‐MSCs treatment effectively attenuates surgery‐induced neuroinflammation.

### 3.6 hUC‐MSCs Modulate the Surgery‐Induced STMN2/SARM1/NF‐κB Signaling Pathway

**FIGURE 5:**
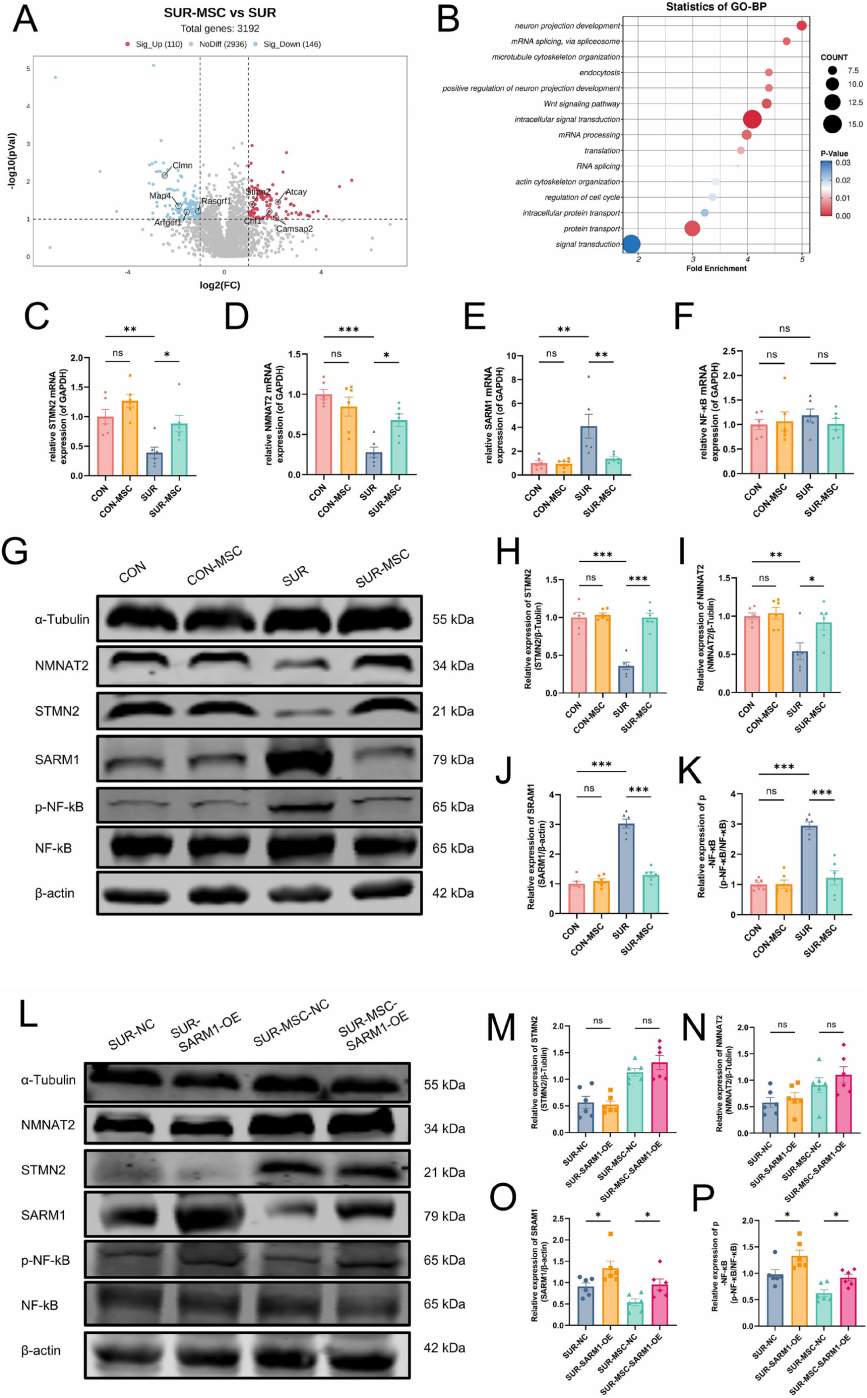
hUC‐MSCs modulate the surgery‐induced STMN2/NMNAT2‐SARM1‐NF‐κB signaling pathway. (A) Volcano plot illustrating differential protein expression in hippocampal tissues between the SUR group and SUR‐MSC group, with thresholds set at |log2(fold change)| > 1 and *p*‐value < 0.1. (B) Dot plot showing the top 15 enriched biological processes from GO‐BP analysis of differentially expressed proteins. Dot sizes correspond to the number of proteins identified for each term, while colors represent *p*‐values. qPCR analysis of hippocampal STMN2 (C), NMNAT2 (D), SARM1 (E), and NF‐κB (F) gene expression (*n* = 6). Representative Western blot bands (G) and quantitative analysis of STMN2 (H), NMNAT2 (I), SARM1 (J), and p‐NF‐κB/NF‐κB (K) protein expression in hippocampal tissues from CON, CON‐MSC, SUR, and SUR‐MSC groups (*n* = 6). Representative Western blot bands (L) and quantitative analysis of STMN2 (M), NMNAT2 (N), SARM1 (O), and p‐NF‐κB/NF‐κB (P) protein expression in hippocampal tissues from SUR‐NC, SUR‐SARM1‐OE, SUR‐MSC‐NC, and SUR‐MSC‐SARM1‐OE groups (*n* = 6). Data are presented as mean ± SEM. *p < 0.05, **p < 0.01, ***p < 0.001.

Next, to investigate how hUC‐MSCs modulate surgery‐induced neuroinflammation, we performed proteomic sequencing analysis through OmicStudio. Under the screening criteria of *p* < 0.1 and fold change ≥ 2 or ≤ −2 for statistically significant differences, 256 differentially expressed proteins (DEPs) were identified in the SUR‐MSC group compared to the SUR group, including 110 significantly upregulated proteins and 146 significantly downregulated proteins (Figure 5A).

To explore proteins closely associated with the CNS, GO‐BP analysis revealed that the DEPs were primarily enriched in neuronal projection development (Figure 5B). Among these, four were upregulated (Atcay, Camsap2, Chl1, and STMN2), while four were downregulated (Clmn, MAP4, Arfgef1, and Rasgrf1). The functional characterization of Atcay, Camsap2, Chl1, and Clmn, along with their related pathways in neuroinflammatory processes and cognitive regulation, remains poorly understood in current literature. Given the low relevance of these four DEPs to PND, they were excluded. The remaining DEPs, whose associated pathways have been reported in neuroinflammation or cognition, were further validated via qPCR. Among them, MAP4, Arfgef1, and Rasgrf1 showed no significant intergroup differences (Figure S4A). Notably, studies have indicated that STMN2, a neuronal axonal survival factor, cooperates with NMNAT2 to regulate downstream SARM1 [19, 41]. Subsequent Western blot and qPCR analyses of mouse hippocampal tissues revealed that, compared to the CON group, the SUR group exhibited downregulated STMN2 and NMNAT2 alongside upregulated SARM1, while hUC‐MSCs treatment reversed these changes (Figure 5C–E,G–J). Furthermore, research suggests that the NF‐κB signaling pathway is a major downstream effector of SARM1 activation. qPCR analysis of mouse hippocampal tissues revealed no statistically significant differences in total NF‐κB mRNA expression levels (Figure 5F). Since phosphorylated NF‐κB (p‐NF‐κB) represents the activated form of this pathway, we performed Western blot analysis, which showed significantly elevated p‐NF‐κB/NF‐κB ratios in the SUR group compared to CON controls, which was reversed by hUC‐MSCs treatment (Figure 5G,K).

To validate the relationships within this pathway, we stereotactically injected HBAAV2/9‐m‐Sarm1‐3xflag‐mcherry (to overexpress SARM1 in the hippocampal CA1 region) or HBAAV2/9‐CMV‐mCherry (negative control) into the mouse brains. The HBAAV2/9‐Sarm1‐3xflag‐mcherry construct effectively overexpressed SARM1 in the hippocampal CA1 region, as confirmed by fluorescence imaging, qPCR, and Western blot analysis (Figure S4B–E). After 28 days of viral infection and model establishment, hippocampal tissues were subjected to western blot analysis using the same experimental procedures. The results demonstrated no significant differences in STMN2 and NMNAT2 protein expression between PND‐NC and PND‐SARM1‐OE groups, nor between PND‐MSC‐NC and PND‐MSC‐SARM1‐OE groups. However, compared to the PND‐NC group, the PND‐SARM1‐OE group showed significantly upregulated SARM1 and p‐NF‐κB/NF‐κB expression. Similarly, the PND‐MSC‐SARM1‐OE group exhibited significantly higher SARM1 and p‐NF‐κB/NF‐κB levels than the PND‐MSC‐NC group (Figure 5L–P). These findings indicate that SARM1 overexpression acts downstream of STMN2 and NMNAT2 without regulating their expression, while functioning as an upstream regulator of the NF‐κB signaling pathway. This confirms the STMN2/NMNAT2‐SARM1‐NF‐κB cascade and identifies SARM1 as the key target through which hUC‐MSCs modulate neuroinflammation in a PND mouse model.

Additionally, immunofluorescence staining was performed to determine SARM1 expression in different cell types in the hippocampal CA1 region. The results showed that SARM1 (orange fluorescence) was highly expressed in neurons (purple fluorescence) but was minimally detected in microglia (cyan fluorescence) and astrocytes (green fluorescence) (Figure S4F).

These results demonstrated that surgical intervention downregulated STMN2 and NMNAT2 expression in the hippocampus, while hUC‐MSCs treatment effectively reversed this downregulation. Furthermore, hUC‐MSCs suppressed SARM1 and p‐NF‐κB expression in neurons, thereby ameliorating neuroinflammation.

### 3.7 Overexpression of SARM1 Reverse the Therapeutic Effects of hUC‐MSCs

**FIGURE 6:**
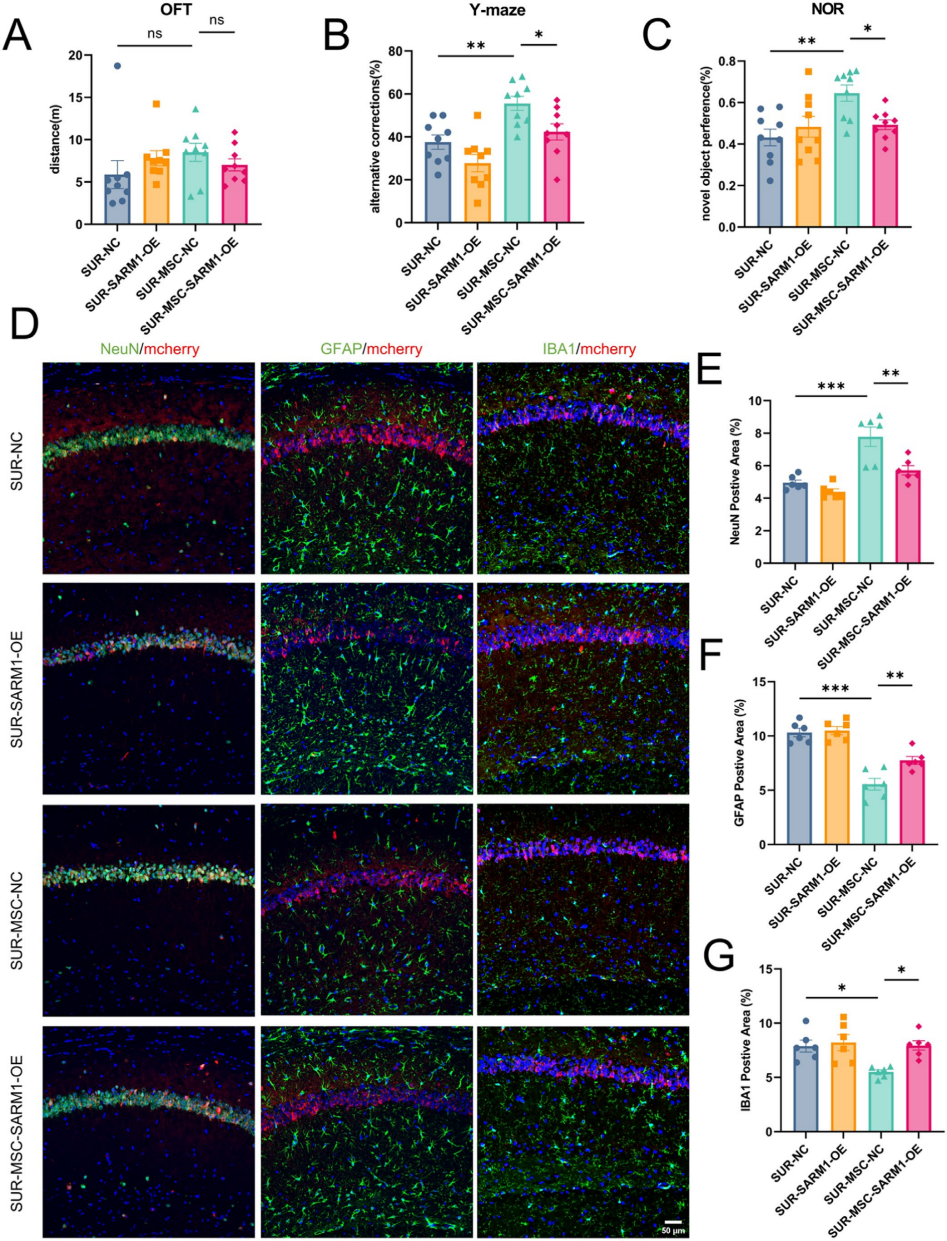
Overexpression of SARM1 reverses the therapeutic effects of hUC‐MSCs. (A) Total distance traveled by mice in OFT (*n* = 9). (B) Alternative corrections (%) in Y‐maze test (*n* = 9). (C) Novel object preference (%) in NOR (*n* = 9). (D) Representative GFAP, NeuN, and IBA1 immunostaining images of the hippocampal CA1 region. (E) Quantitative analysis of the percentage of NeuN positive area in the hippocampal CA1 region (*n* = 6). (F) Quantitative analysis of the percentage of GFAP positive area in the hippocampal CA1 region (*n* = 6). (G) Quantitative analysis of the percentage of IBA1 positive area in the hippocampal CA1 region (*n* = 6). Data are presented as mean ± SEM. *p < 0.05, **p < 0.01, ***p < 0.001.

To determine whether hUC‐MSCs improve cognitive impairment by downregulating hippocampal SARM1 expression postoperatively, we followed the same experimental protocol and injected either HBAAV2/9‐m‐Sarm1‐3xflag‐mcherry or HBAAV2/9‐CMV‐mcherry into the bilateral hippocampal CA1 regions to overexpress SARM1. After 28 days, anesthesia and surgery were performed, followed by behavioral tests on postoperative Day 3. The results showed no significant differences in total distance moved or movement velocity among the four groups, confirming that motor function remained unaffected (Figure 6A and Figure S5). While no significant differences were observed in the correct alternation rate or novel object exploration time between the PND‐NC and PND‐SARM1‐OE groups, the PND‐MSC‐SARM1‐OE group exhibited decreased performance in both parameters compared to the PND‐MSC‐NC group (Figure 6B,C and Figure S4).

To further investigate the role of SARM1 in neuronal apoptosis, NeuN^+^ positive area analysis revealed significantly increased neuronal apoptosis in the PND‐SARM1‐OE group compared to the PND‐NC group, with similarly elevated apoptosis observed in the PND‐MSC‐SARM1‐OE group relative to the PND‐MSC‐NC group. Concurrent assessment of SARM1's effect on neuroinflammation through IBA‐1^+^ and GFAP^+^ positive area quantification demonstrated exacerbated neuroinflammatory responses in both SARM1‐OE groups compared to their respective controls (Figure 6D–G).

These findings collectively demonstrated that SARM1 overexpression effectively counteracted the therapeutic benefits of hUC‐MSCs on cognitive function, neuronal apoptosis, and neuroinflammation, thereby establishing SARM1 as a pivotal mediator in hUC‐MSCs‐based treatment of perioperative neurocognitive disorders.

### 3.8 hUC‐MSCs Regulate Postoperative SARM1‐Mediated Dendritic Degeneration

**FIGURE 7:**
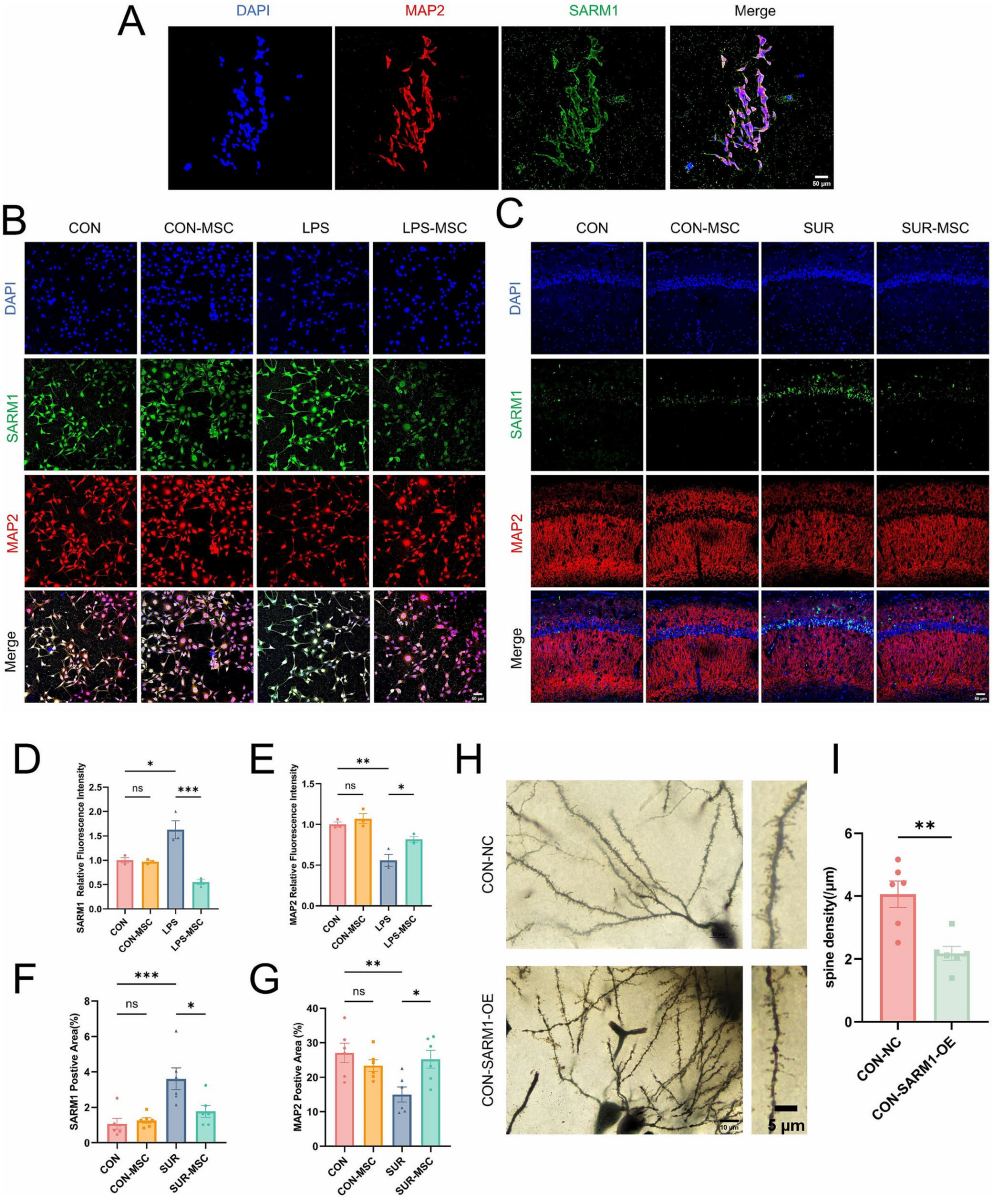
hUC‐MSCs regulate postoperative SARM1‐mediated dendritic degeneration. (A) Representative co‐localization images of MAP2 and SARM1 double immunostaining in HT22 cells. (B) Immunofluorescence staining of MAP2 and SARM1 in HT22 cells under different treatment conditions. (C) Immunofluorescence staining of MAP2 and SARM1 in the hippocampal CA1 region. (D, E) Quantitative analysis of the relative fluorescence intensity of MAP2 and SARM1 in HT22 cells (*n* = 3). (F, G) Quantitative analysis of the positive area of MAP2 and SARM1 in the hippocampal CA1 region (*n* = 6). (H) Representative Golgi staining images of the hippocampal CA1 region. (I) Quantitative analysis of dendritic spine density in the hippocampal CA1 region (*n* = 6). Data are presented as mean ± SEM. *p < 0.05, **p < 0.01, ***p < 0.001.

We observed that PND led to a decrease in dendritic spine density. Given the existing evidence identifying SARM1 as a key regulator of dendritic morphology [22], we proposed the hypothesis that hUC‐MSCs upregulate dendritic spine density through SARM1 modulation. In vitro, HT‐22 cells demonstrated strong co‐localization between SARM1 and MAP2 (a dendrite marker) through double immunofluorescence staining (Figure 7A). Compared to the CON group, LPS‐treated cells showed upregulated SARM1 expression and downregulated MAP2 levels, while the LPS‐MSC group exhibited reversed expression patterns with decreased SARM1 and increased MAP2 levels (Figure 7B,D,E). These in vitro findings were consistently replicated in the hippocampal CA1 region (Figure 7C,F,G).

To further investigate SARM1's role in dendritic degeneration, we performed bilateral hippocampal CA1 injections of either HBAAV2/9‐m‐Sarm1‐3xflag‐mcherry or control HBAAV2/9‐CMV‐mcherry (following the same protocol) to overexpress SARM1. Without anesthesia/surgical intervention, Golgi‐Cox staining performed 28 days postinjection revealed significantly reduced dendritic spine density in the CON‐SARM1‐OE group compared to that in the CON‐NC controls (Figure 7H,I).

These results demonstrated that SARM1 played a crucial role in mediating dendritic degeneration, while hUC‐MSCs ameliorate surgery‐induced dendrite pathology in the hippocampal CA1 region through SARM1 regulation.

### 3.9 hUC‐MSCs Exert Therapeutic Effects in the PND Mouse Model via β‐NGF

**FIGURE 8:**
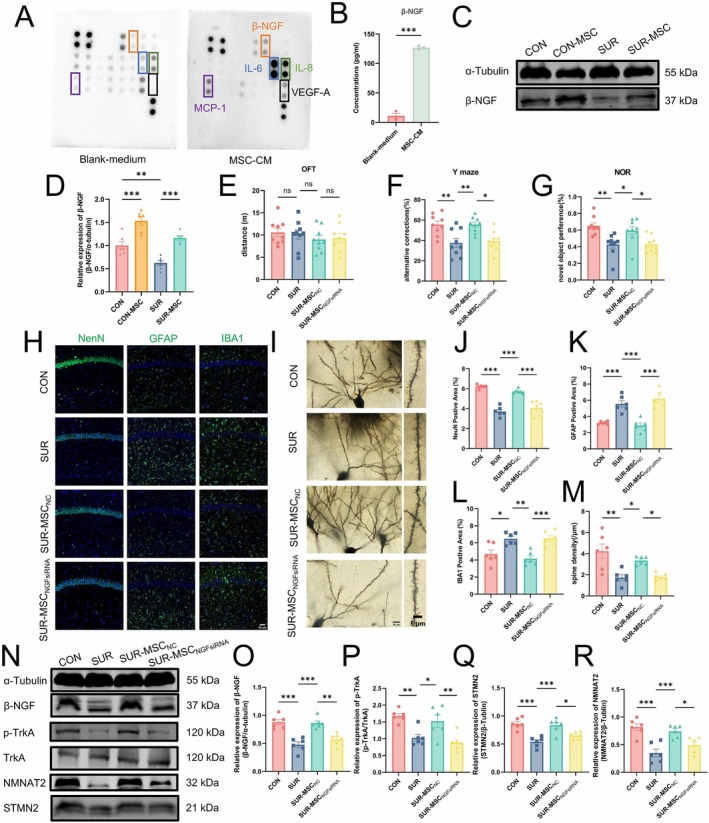
hUC‐MSCs exert therapeutic effects in the PND mouse model via β‐NGF. (A) 20‐Human Neuro Discovery array analysis between blank‐medium and MSC‐conditioned medium (MSC‐CM) (B) ELISA quantification of β‐NGF levels in blank‐medium and MSC‐CM (*n* = 3). (C, D) Representative Western blot bands and quantitative analysis of β‐NGF protein expression in hippocampal tissues from CON, CON‐MSC, SUR, and SUR‐MSC groups. (E) Total distance traveled by mice in OFT (*n* = 9). (F) Alternative corrections (%) in Y‐maze test (*n* = 9). (G) Novel object preference (%) in NOR (*n* = 9). (H) Representative GFAP, NeuN and IBA1 immunostaining images of hippocampal CA1 region. (I) Representative Golgi staining images (J) Quantitative analysis of NeuN positive area percentage in hippocampal CA1 region (*n* = 6). (K) Quantitative analysis of GFAP positive area percentage in hippocampal CA1 region (*n* = 6). (L) Quantitative analysis of IBA1 positive area percentage in hippocampal CA1 region (*n* = 6). (M) Quantitative analysis of dendritic spine density in hippocampal CA1 region. Representative Western blot bands (N) and quantitative analysis of β‐NGF (O), p‐TrkA/TrkA (P), STMN2 (Q) and NMNAT2 (R) protein expression in hippocampal tissues from CON, SUR, SUR‐MSC_NC_ and SUR‐MSC_NGFsiRNA_ groups (*n* = 6). Data are presented as mean ± SEM. *p < 0.05, **p < 0.01, ***p < 0.001.

To investigate the specific factors through which hUC‐MSCs exert their cognitive‐improving effects, we analyzed 20 neuro‐related factors in blank control medium versus hUC‐MSC‐conditioned medium using antibody arrays. The results revealed that hUC‐MSCs secreted five key factors: the neurotrophic factor β‐NGF, VEGF‐A, and the chemokines IL‐6, IL‐8, and MCP‐1 (Figure 8A and Figure S6A). Existing evidence suggests that β‐NGF, through its binding to TrkA, plays the most direct role in cognitive function and neuronal repair, and appears to regulate STMN2 expression [32, 42, 43]. We therefore hypothesized that β‐NGF is critically involved in PND pathogenesis. ELISA confirmed significantly elevated β‐NGF levels in hUC‐MSC‐conditioned medium compared to blank control (Figure 8B). Western blot analysis demonstrated downregulated hippocampal β‐NGF in PND mice versus controls, which was partially restored by hUC‐MSCs treatment (Figure 8C,D). However, it remains unclear whether the increased β‐NGF originates from direct secretion by hUC‐MSCs or indirect induction in host mice mediated by hUC‐MSCs. To distinguish these two possibilities, we performed species‐specific ELISA assays to separately quantify mouse and human β‐NGF. Compared with the CON group, the CON‐MSC group exhibited no significant difference in mouse β‐NGF but a marked upward trend in human β‐NGF. Similarly, relative to the SUR group, the SUR‐MSC group showed no significant alteration in mouse β‐NGF yet a pronounced increase in human β‐NGF. These results indicate that the elevated hippocampal β‐NGF protein levels are derived primarily from direct secretion by transplanted hUC‐MSCs (Figure S6B). To validate β‐NGF as the key therapeutic target, we employed siRNA to knock down β‐NGF expression in hUC‐MSCs, with successful inhibition confirmed by Western blot and PCR (Figure S6C–E). In behavioral assessments (OFT, Y‐maze, NOR), the SUR‐MSC_NC_ group showed improved cognitive function relative to the SUR group, while SUR‐MSC_NGFsiRNA_ treatment abolished these beneficial effects (Figure 8E–G and Figure S6F). Immunofluorescence analysis revealed no significant differences in GFAP, IBA‐1, or NeuN‐positive areas between SUR and SUR‐MSC_NGFsiRNA_ groups, whereas SUR‐MSC_NGFsiRNA_ reversed the neuroprotective and anti‐inflammatory effects of MSC_NC_ (Figure 8H,J–L). Golgi‐Cox staining indicated that SUR‐MSC_NGFsiRNA_ failed to rescue dendritic spine density compared to the SUR group, and counteracted the spine improvement mediated by MSC_NC_ (Figure 8I,M). Western blot analysis revealed a significant decrease in β‐NGF levels and its receptor p‐TrkA/TrkA ratio in the SUR group compared to CON controls, which was restored by MSC_NC_ treatment. This restoration was effectively reversed in the SUR‐MSC_NGFsiRNA_ group (Figure 8N–P). Furthermore, the SUR‐MSC_NGFsiRNA_ group failed to upregulate STMN2 and NMNAT2 protein expression compared to the SUR group and it significantly attenuated the MSC_NC_‐induced upregulation of these neuroprotective proteins (Figure 8N,Q,R). To verify the direct effect of β‐NGF on neurons, we treated HT‐22 cells with recombinant β‐NGF or GW‐441756. The CCK‐8 assay showed that 50 ng/mL β‐NGF and 100 nM GW‐441756 exhibited no cytotoxicity in HT‐22 cells, and β‐NGF exerted a moderate pro‐proliferative effect on this cell line (Figure S6G). Furthermore, Western blot analysis revealed that GW‐441756 treatment significantly reduced NMNAT2 and STMN2 protein levels compared with the Vehicle group, whereas β‐NGF treatment significantly increased their expression. Notably, the β‐NGF‐induced upregulation of NMNAT2 and STMN2 was suppressed by co‐treatment with GW‐441756 (Figure S6H,I).

These results demonstrated that β‐NGF is indispensable for hUC‐MSCs‐mediated cognitive improvement, neuroprotection against apoptosis, attenuation of neuroinflammation, restoration of dendritic spine integrity, and upregulation of STMN2 and NMNAT2 protein levels, thereby establishing β‐NGF as the pivotal neurotrophic factor underlying hUC‐MSCs' therapeutic efficacy in PND treatment.

## 4 Discussion

This study demonstrated that tibial fracture intramedullary fixation can induce learning and memory impairments in mice, accompanied by pathological changes including hippocampal neuronal apoptosis, neuroinflammation, and dendritic degeneration. Transplantation of hUC‐MSCs significantly ameliorated these pathological manifestations through modulation of the STMN2/NMNAT2‐SARM1‐NF‐κB signaling pathway in PND treatment. Furthermore, through cytokine array analysis and functional interference experiments, we have for the first time identified β‐NGF as a key effector molecule mediating hUC‐MSCs‐induced improvement in PND mice.

Systemic inflammatory responses triggered by surgical trauma can propagate to the CNS, leading to PND through neuroinflammation. This consequently prolongs hospitalization, increases healthcare burden, and may progress to chronic neurodegenerative diseases such as Alzheimer's disease [1, 44, 45, 46]. Therefore, effective PND treatment remains a critical unmet medical need. Although interventions like red light therapy, upconversion nanoparticles, and electroacupuncture have demonstrated excellent efficacy in animal models [47, 48, 49], their operational complexity limits clinical translation. Over the past decades, no breakthrough has been achieved in monotherapy for PND, while hUC‐MSCs offer a novel cell‐based therapeutic approach distinct from conventional pharmacotherapy. Previous studies have demonstrated that MSCs can ameliorate cognitive impairment in PND and have explored the underlying mechanisms [50]. However, the in vivo distribution of hUC‐MSCs in PND remains unclear, and it is still unknown which specific substances secreted by hUC‐MSCs—as complex cellular entities—exert this therapeutic effect. Furthermore, we have explored novel mechanisms for PND amelioration and conducted further research into the causes of dendritic degeneration.

Research on stem cell therapy has been expanding rapidly. hUC‐MSCs have been shown to improve cognitive function in AD through HGF [14]. The combination of hUC‐MSCs with resveratrol promotes neural repair in AD [51]. Coordinated effects of electrical stimulation and MSCs alleviate inflammatory status and elevate dopamine levels in PD [52], while hUC‐MSCs exhibit anti‐inflammatory effects and restore motor function in Amyotrophic Lateral Sclerosis (ALS) mice [53]. In Huntington's disease models, hUC‐MSCs downregulate pp65 levels while reducing GFAP proliferation and inflammation. Clinical trials with MSCs are currently underway, including the first FDA‐approved Phase I/II trial (NCT02833792) of intraventricular MSC administration for mild‐to‐moderate AD, which has demonstrated safety and is evaluating long‐term clinical efficacy [54]. Another study involving 7 PD patients receiving infusions of MSCs into the subventricular zone confirmed both safety and significant cognitive improvement [55]. Clinical investigations are also ongoing for spinal cord injury (SCI) and ALS [56, 57]. Although numerous studies have demonstrated MSC‐mediated improvements in neuroinflammation, neuronal apoptosis, and dendritic spine alterations across various cognition‐related disorders [58, 59, 60], research on MSCs for PND remains scarce, particularly regarding the specific mediators underlying cognitive improvement.

Therefore, we conducted this investigation of hUC‐MSCs in PND mice. Our tibial fracture intramedullary fixation model was established based on previous literature and PND pathological mechanisms [61, 62]. This surgical procedure induces systemic inflammation, which has been extensively documented to cause cognitive impairment [63, 64]. We successfully established the PND model and confirmed that hUC‐MSCs effectively improved cognitive dysfunction in PND mice. Subsequent investigations focused on whether hUC‐MSCs act directly within the CNS or through peripheral organs. Our findings revealed blood–brain barrier disruption in PND mice, consistent with Ailin Luo's study [65]. Studies have confirmed that NF‐κB signaling alterations and microglial activation can induce BBB damage [66, 67], which correlates with our observation of surgical activation of NF‐κB signaling and microglia. This suggests combined effects of NF‐κB signaling and microglial activation may underlie BBB impairment, explaining the limited hUC‐MSCs presence in brain tissue. Additionally, pro‐inflammatory cytokines (IL‐6, IL‐1β, TNF‐α) represent important mechanisms contributing to BBB damage [68, 69]. Our study found hUC‐MSCs primarily localized in the liver with a small number distributed in the brain, leading us to propose that hUC‐MSCs may exert therapeutic effects through a peripheral‐central synergistic mechanism involving hepatic paracrine signaling and direct cerebral actions.

The pathological mechanisms underlying PND remain incompletely understood. Neuronal apoptosis represents a core pathological component of PND [70, 71], as surgical stress disrupts the balance between pro‐ and antisapoptotic proteins, leading to neuronal apoptosis and cognitive deficits [72]. MSCs and their secretome have been demonstrated to promote neurogenesis and restore dendritic density in AD and PD models [73, 74, 75]. Our study confirms that hUC‐MSCs similarly reduce neuronal apoptosis, exert neuroprotective effects, decrease dendritic degeneration, and increase spine density in PND. Furthermore, as crucial immune cells in the CNS, microglia play decisive roles in neuroinflammatory processes. MSCs possess the capacity to shift microglia from pro‐inflammatory to anti‐inflammatory states, thereby protecting against neural damage [76]. Our results show that hUC‐MSCs not only reduced microglial activation but also decreased M1 pro‐inflammatory microglia while increasing M2 anti‐inflammatory populations, highlighting their immunomodulatory capabilities.

Subsequent proteomic sequencing revealed that hUC‐MSCs‐mediated cognitive improvement in PND mice was associated with upregulated hippocampal STMN2 expression. Correspondingly, we observed coordinated alterations in STMN2, NMNAT2, and downstream SARM1/NF‐κB signaling in hUC‐MSCs‐treated PND mice. As known inhibitors of SARM1 activation [19], the neurite growth‐associated proteins STMN2 and NMNAT2 play regulatory roles. Although extensively studied in various neural injuries as a TLR adaptor protein family member, SARM1's role in PND remains unclear. Growing evidence indicates multifaceted functions of SARM1 in brain development, influencing both apoptosis and dendritic degeneration [20, 22, 77]. Since synaptic formation is inseparable from dendrites and axons, with dendritic spines playing essential roles in synaptic connections, our study confirmed predominant neuronal localization of SARM1 with high co‐localization with dendritic spines. PND was associated with SARM1 upregulation, and hippocampal SARM1 overexpression counteracted hUC‐MSCs neuroprotection, suggesting neuronal SARM1 as a critical mediator of apoptosis and dendritic pathology in PND mice. Additionally, SARM1 is closely associated with neuroinflammation, particularly NF‐κB signaling changes. Studies have confirmed NF‐κB as a major downstream pathway of SARM1 [78, 79]. In spinal cord injury models, SARM1‐knockdown can reduce NF‐κB activity and attenuate neuroinflammation [80, 81]. Combined with our finding that SARM1 overexpression negates hUC‐MSCs anti‐neuroinflammatory effects, we propose this signaling pathway as a key mechanism underlying hUC‐MSCs‐mediated neuroprotection and CNS inflammation suppression.

NGF plays a crucial role in nervous system development, demonstrating essential functions in neuronal survival and dendritic remodeling while exhibiting significant anti‐inflammatory effects [82, 83]. In this study, we observed markedly reduced NGF expression in the hippocampal region of PND model mice. Administration of hUC‐MSCs with NGF knockdown abrogated the therapeutic effects on ameliorating cognitive dysfunction, neuronal apoptosis, and neuroinflammatory responses, as well as rescuing dendritic degeneration and restoring the expression levels of STMN2 and NMNAT2. Furthermore, in vitro experiments using NGF and GW‐441756 confirmed the direct effect of NGF on neurons: NGF not only upregulated the levels of STMN2 and NMNAT2 but also promoted the proliferation of HT‐22 cells. These findings provide novel evidence that NGF is the key effector molecule underlying the therapeutic efficacy of hUC‐MSCs in the treatment of PND. Interestingly, while multiple studies have established the anti‐inflammatory properties of hUC‐MSCs, our neurofactor array analysis revealed upregulated IL‐6 expression in hUC‐MSCs conditioned medium. However, in vivo experiments demonstrated significantly decreased IL‐6 levels in brain tissues following hUC‐MSCs treatment. This apparent paradox may be explained by the dual functionality of IL‐6: during early phases, hUC‐MSCs‐derived IL‐6 may transiently increase and bind to membrane‐bound IL‐6 receptors, promoting recruitment and activation of immune cells including T cells, B cells, and macrophages to initiate anti‐inflammatory pathways. In later stages, the predominant effects of other anti‐inflammatory factors may lead to overall IL‐6 downregulation [84, 85]. Furthermore, our neurofactor array analysis detected significant upregulation of NGF but not BDNF levels, differing from findings reported by Zhi‐Yuan Guo [86]. This discrepancy suggests that culture conditions can substantially influence hUC‐MSCs secretory profiles. Supporting this notion, previous studies have demonstrated that hypoxic conditions enhance TGF‐β secretion by MSCs [59]. Therefore, future studies should focus on optimizing culture conditions to further augment hUC‐MSCs NGF secretion capacity and enhance therapeutic efficacy against PND.

This study holds significant clinical implications. From the perspective of hUC‐MSCs‐based drug development, hUC‐MSCs present several advantages: they raise fewer ethical concerns, are readily available, and can be easily standardized for culture and expansion. Furthermore, future engineering modifications could enhance hUC‐MSCs to secrete more β‐NGF or other therapeutic molecules to improve cognitive function in PND patients. On the other hand, β‐NGF as a promising therapeutic target, warrants future investigation into small‐molecule drugs that can be administered via minimally invasive routes (e.g., intravenous injection or nebulized inhalation) to enhance cognitive function through targeted brain delivery. Additionally, the potential synergistic effects between small‐molecule drugs and hUC‐MSCs could be explored to enhance therapeutic outcomes.

## 5 Conclusion

In summary, PND leads to cognitive dysfunction, neuronal apoptosis, neuroinflammation, and dendritic degeneration in mice. hUC‐MSCs can exert ameliorating effects. hUC‐MSCs secrete β‐NGF, activate TrkA signaling, upregulate STMN2 and NMNAT2, and inhibit the SARM1/NF‐κB axis. Overexpression of SARM1 or knockdown of β‐NGF reverses its therapeutic effects, suggesting a potential cell‐based therapeutic strategy for PND, with future research directions including optimizing hUC‐MSCs' secretory function or developing β‐NGF‐targeted small‐molecule drugs.

## Author Contributions

Before initiating the experiment, we developed the experimental plan in detail and followed it rigorously. The conception and design of the study were carried out by K.W. and F.W. K.W., Y.Z., and F.W. collected and analyzed the data. K.W., F.W., Y.Z., W.L., S.L., Y.Z., G.M., and Z.X. performed the experiments. X.Z. and L.W. wrote the manuscript. All authors read through and approved the final draft of the manuscript.

## Funding

National Natural Science Foundation of China, 81700078; Xuzhou Medical Key Talent Project, XWRCHT20220051; Foundation of Jiangsu Provincial Health Commission, M2024008; Project of Jiangsu Provincial Key Laboratory of New Drug Research and Clinical Pharmacy, XZSYSKF2024020; Concept Validation Project of Xuzhou Medical University, GNYZ2024006.

## Ethics Statement

All efforts were made to minimize the number and suffering of the animals used in the study. The experimental protocol was approved by the Xuzhou Animal Care and Use Committee (202,504 T033) and was conducted in accordance with the Institutional Animal Care and Use Guidelines of Xuzhou Medical University.

## Conflicts of Interest

The authors declare no conflicts of interest.

## Supporting information


**Figure S1:** Representative X‐ray image of tibial fracture intramedullary fixation.
**Figure S2:** Characteristics and differentiation potential of clinical‐grade hUC‐MSCs. (A) hUC‐MSCs exhibited characteristic fibroblast‐like morphology at passage 5. (B–D) Differentiation capacities of hUC‐MSCs into osteocytes, chondrocytes and adipocytes. Osteogenic differentiation was indicated by the formation of a mineralized matrix (shown by staining with Alizarin Red). Adipogenic differentiation was demonstrated by the formation of fat droplets positively stained with Oil red O. Chondrogenic differentiation was verified by the presence of proteoglycans stained with Alcian Blue. (E–I) Flow cytometric analysis showed that hUC‐MSCs were positive for CD29, CD44, CD90, but negative for CD34 and CD45.
**Figure S3:** hUC‐MSCs alleviated surgery‐induced neuroinflammation, related to Figure 4. (A) Hippocampal tissues were collected 24 h post‐surgery for ELISA measurement of IL‐1β, IL‐6, and TNF‐α levels. (B, C) Representative IBA1 and GFAP immunostaining images and quantitative analysis of the percentage of IBA1‐ and GFAP‐positive area of the hippocampal CA1 region (*n* = 6). (D, E) Representative IBA1 and GFAP immunostaining images and quantitative analysis of the percentage of IBA1‐ and GFAP‐positive area of the hippocampal CA3 region (*n* = 6). (F, G) Representative IBA1 and GFAP immunostaining images and quantitative analysis of the percentage of IBA1‐ and GFAP‐positive area of the hippocampal DG region (*n* = 6). Data are presented as mean ± SEM. *p < 0.05, **p < 0.01, ***p < 0.001.
**Figure S4:** Differential gene expression in hippocampal tissues and validation of AAV‐mediated SARM1 overexpression, related to Figure 5. (A) qPCR analysis of hippocampal Rasgrf1, Arfgef1, and MAP4 gene expression (*n* = 6). (B) Fluorescence images demonstrating effective AAV vector expression in the hippocampal CA1 region. (C) qPCR analysis of hippocampal SARM1 expression (*n* = 6). Representative Western blot bands (D) and quantitative analysis of SARM1 protein expression (E) in hippocampal tissues from CON‐NC and CON‐SARM1‐OE groups (*n* = 6). (F) Multiplex immunostaining of SARM1 (orange), NeuN (purple), GFAP (green) and IBA‐1 (cyan). Data are presented as mean ± SEM. *p < 0.05, **p < 0.01.
**Figure S5:** Representative movement trajectories of mice in OFT, Y‐maze, and NOR, related to Figure 6.
**Figure S6:** Representative movement trajectories of mice in OFT, Y‐maze, and NOR and validation of siRNA‐mediated β‐NGF knockdown, related to Figure 8. (A) The corresponding protein names and their positions on the Human Neuro Discovery array shown in the table. (B) Hippocampal tissues were collected 24 h post‐surgery for ELISA measurement of human, mouse and total β‐NGF levels. (C) qPCR analysis of β‐NGF gene expression (*n* = 3). Representative Western blot bands(D) and (E) quantitative analysis of β‐NGF protein expression from MSCNC and MSCNGFsiRNA groups (*n* = 3). (F) Representative movement trajectories of mice in OFT, Y‐maze, and NOR. (G) Cell viability was measured by CCK‐8 assay after treatment with NGF or GW‐441756. (H, I) Representative Western blot bands and quantitative analysis of NMNAT2 and STMN2 protein expression in hippocampal tissues. Data are presented as mean ± SEM. *p < 0.05, **p < 0.01, ***p < 0.001.

## Data Availability

The data that support the findings of this study are available from the corresponding author upon reasonable request.
