# Supplementary material for: hUC‐MSCs via β‐NGF Alleviate Cognitive Impairment After Tibial Fracture Surgery by Regulating the STMN2/NMNAT2‐SARM1‐NF‐κB Signaling Pathway
Source: CNS Neurosci Ther. 2026 Jul 29;32(8):e71062. doi: 10.1002/cns.71062 (PMC13418377; doi:10.1002/cns.71062)
Supplement: Supplementary file 1 — Figure S1: Representative X‐ray image of tibial fracture intramedullary fixation. Figure S2: Characteristics and differentiation potential of clinical‐grade hUC‐MSCs. (A) hUC‐MSCs exhibited characteristic fibroblast‐like morphology at passage 5. (B–D) Differentiation capacities of hUC‐MSCs into osteocytes, chondrocytes and adipocytes. Osteogenic differentiation was indicated by the formation of a mineralized matrix (shown by staining with Alizarin Red). Adipogenic differentiation was demonstrated by the formation of fat droplets positively stained with Oil red O. Chondrogenic differentiation was verified by the presence of proteoglycans stained with Alcian Blue. (E–I) Flow cytometric analysis showed that hUC‐MSCs were positive for CD29, CD44, CD90, but negative for CD34 and CD45. Figure S3: hUC‐MSCs alleviated surgery‐induced neuroinflammation, related to Figure 4. (A) Hippocampal tissues were collected 24 h post‐surgery for ELISA measurement of IL‐1β, IL‐6, and TNF‐α levels. (B, C) Representative IBA1 and GFAP immunostaining images and quantitative analysis of the percentage of IBA1‐ and GFAP‐positive area of the hippocampal CA1 region (n = 6). (D, E) Representative IBA1 and GFAP immunostaining images and quantitative analysis of the percentage of IBA1‐ and GFAP‐positive area of the hippocampal CA3 region (n = 6). (F, G) Representative IBA1 and GFAP immunostaining images and quantitative analysis of the percentage of IBA1‐ and GFAP‐positive area of the hippocampal DG region (n = 6). Data are presented as mean ± SEM. *p < 0.05, **p < 0.01, ***p < 0.001. Figure S4: Differential gene expression in hippocampal tissues and validation of AAV‐mediated SARM1 overexpression, related to Figure 5. (A) qPCR analysis of hippocampal Rasgrf1, Arfgef1, and MAP4 gene expression (n = 6). (B) Fluorescence images demonstrating effective AAV vector expression in the hippocampal CA1 region. (C) qPCR analysis of hippocampal SARM1 expression (n = 6). Representative Western blot bands (D) [file CNS-32-e71062-s001.docx]

**Fig.S1 Representative X‑ray image of tibial fracture intramedullary fixation**





**Fig.S2 Characteristics and differentiation potential of clinical-grade hUC-MSCs. (A)** hUC-MSCs exhibited characteristic fibroblast-like morphology at passage 5. **(B-D)** Differentiation capacities of hUC‑MSCs into osteocytes, chondrocytes and adipocytes. Osteogenic differentiation was indicated by the formation of a mineralized matrix (shown by staining with Alizarin Red). Adipogenic differentiation was demonstrated by the formation of fat droplets positively stained with Oil red O. Chondrogenic differentiation was verified by the presence of proteoglycans stained with Alcian Blue. **(E-I)** Flow cytometric analysis showed that hUC‑MSCs were positive for CD29, CD44, CD90, but negative for CD34 and CD45.








**Fig.S3 hUC-MSCs alleviated surgery-induced neuroinflammation, related to Figure 4. (A)** Hippocampal tissues were collected 24 h post-surgery for ELISA measurement of IL-1β, IL-6, and TNF-α levels. **(B-C)** Representative IBA1 and GFAP immunostaining images and quantitative analysis of the percentage of IBA1- and GFAP-positive area of the hippocampal CA1 region (n=6). **(D-E)** Representative IBA1 and GFAP immunostaining images and quantitative analysis of the percentage of IBA1- and GFAP-positive area of the hippocampal CA3 region (n=6). **(F-G)** Representative IBA1 and GFAP immunostaining images and quantitative analysis of the percentage of IBA1- and GFAP-positive area of the hippocampal DG region (n=6). Data are presented as mean ± SEM. *P<0.05, **P<0.01, ***P<0.001.





**Fig. S4 Differential gene expression in hippocampal tissues and validation of AAV-mediated SARM1 overexpression, related to Figure 5. (A)** qPCR analysis of hippocampal Rasgrf1, Arfgef1, and MAP4 gene expression (n=6). **(B)** Fluorescence images demonstrating effective AAV vector expression in the hippocampal CA1 region. **(C)** qPCR analysis of hippocampal SARM1 expression (n=6). Representative Western blot bands **(D)** and quantitative analysis of SARM1 protein expression **(E)** in hippocampal tissues from CON-NC and CON-SARM1-OE groups (n=6). **(F)** Multiplex immunostaining of SARM1 (orange), NeuN (purple), GFAP (green) and IBA-1 (cyan). Data are presented as mean ± SEM. *P<0.05, **P<0.01.





**Fig. S5 Representative movement trajectories of mice in OFT, Y-maze, and NOR,** **related to Figure 6.**








**Fig. S6 Representative movement trajectories of mice in OFT, Y-maze, and NOR and validation of siRNA-mediated β-NGF knockdown, related to Figure 8. (A)** The corresponding protein names and their positions on the Human Neuro Discovery array shown in the table. **(B)** Hippocampal tissues were collected 24 h post-surgery for ELISA measurement of human, mouse and total β-NGF levels. **(C)** qPCR analysis of β-NGF gene expression (n=3). Representative Western blot bands**(D)** and **(E)** quantitative analysis of β-NGF protein expression from MSC_NC_ and MSC_NGFsiRNA_ groups (n=3). **(F)** Representative movement trajectories of mice in OFT, Y-maze, and NOR. **(G)** Cell viability was measured by CCK-8 assay after treatment with NGF or GW-441756. **(H-I)** Representative Western blot bands and quantitative analysis of NMNAT2 and STMN2 protein expression in hippocampal tissues. Data are presented as mean ± SEM. *P<0.05, **P<0.01, ***P<0.001.
